# Causal inference using observational intensive care unit data: a scoping review and recommendations for future practice

**DOI:** 10.1038/s41746-023-00961-1

**Published:** 2023-11-27

**Authors:** J. M. Smit, J. H. Krijthe, W. M. R. Kant, J. A. Labrecque, M. Komorowski, D. A. M. P. J. Gommers, J. van Bommel, M. J. T. Reinders, M. E. van Genderen

**Affiliations:** 1 Department of Intensive Care, Erasmus University Medical Center, Rotterdam, The Netherlands; 2 Pattern Recognition & Bioinformatics group, EEMCS, Delft University of Technology, Delft, The Netherlands; 3 Data Science group, Institute for Computing and Information Sciences, Radboud University, Nijmegen, The Netherlands; 4 Department of Epidemiology, Erasmus Medical Center, Rotterdam, The Netherlands; 5 Department of Surgery and Cancer, Faculty of Medicine, Imperial College London, London, UK; 6 Intensive Care Unit, Charing Cross Hospital, Imperial College Healthcare NHS Trust, London, UK

**Keywords:** Machine learning, Statistical methods, Epidemiology

## Abstract

This scoping review focuses on the essential role of models for causal inference in shaping *actionable* artificial intelligence (AI) designed to aid clinicians in decision-making. The objective was to identify and evaluate the reporting quality of studies introducing models for causal inference in intensive care units (ICUs), and to provide recommendations to improve the future landscape of research practices in this domain. To achieve this, we searched various databases including Embase, MEDLINE ALL, Web of Science Core Collection, Google Scholar, medRxiv, bioRxiv, arXiv, and the ACM Digital Library. Studies involving models for causal inference addressing *time-varying* treatments in the adult ICU were reviewed. Data extraction encompassed the study settings and methodologies applied. Furthermore, we assessed reporting quality of target trial components (i.e., eligibility criteria, treatment strategies, follow-up period, outcome, and analysis plan) and main causal assumptions (i.e., conditional exchangeability, positivity, and consistency). Among the 2184 titles screened, 79 studies met the inclusion criteria. The methodologies used were G methods (61%) and reinforcement learning methods (39%). Studies considered both static (51%) and dynamic treatment regimes (49%). Only 30 (38%) of the studies reported all five target trial components, and only seven (9%) studies mentioned all three causal assumptions. To achieve actionable AI in the ICU, we advocate careful consideration of the causal question of interest, describing this research question as a target trial emulation, usage of appropriate causal inference methods, and acknowledgement (and examination of potential violations of) the causal assumptions.

## Introduction

**Fig. 1:**
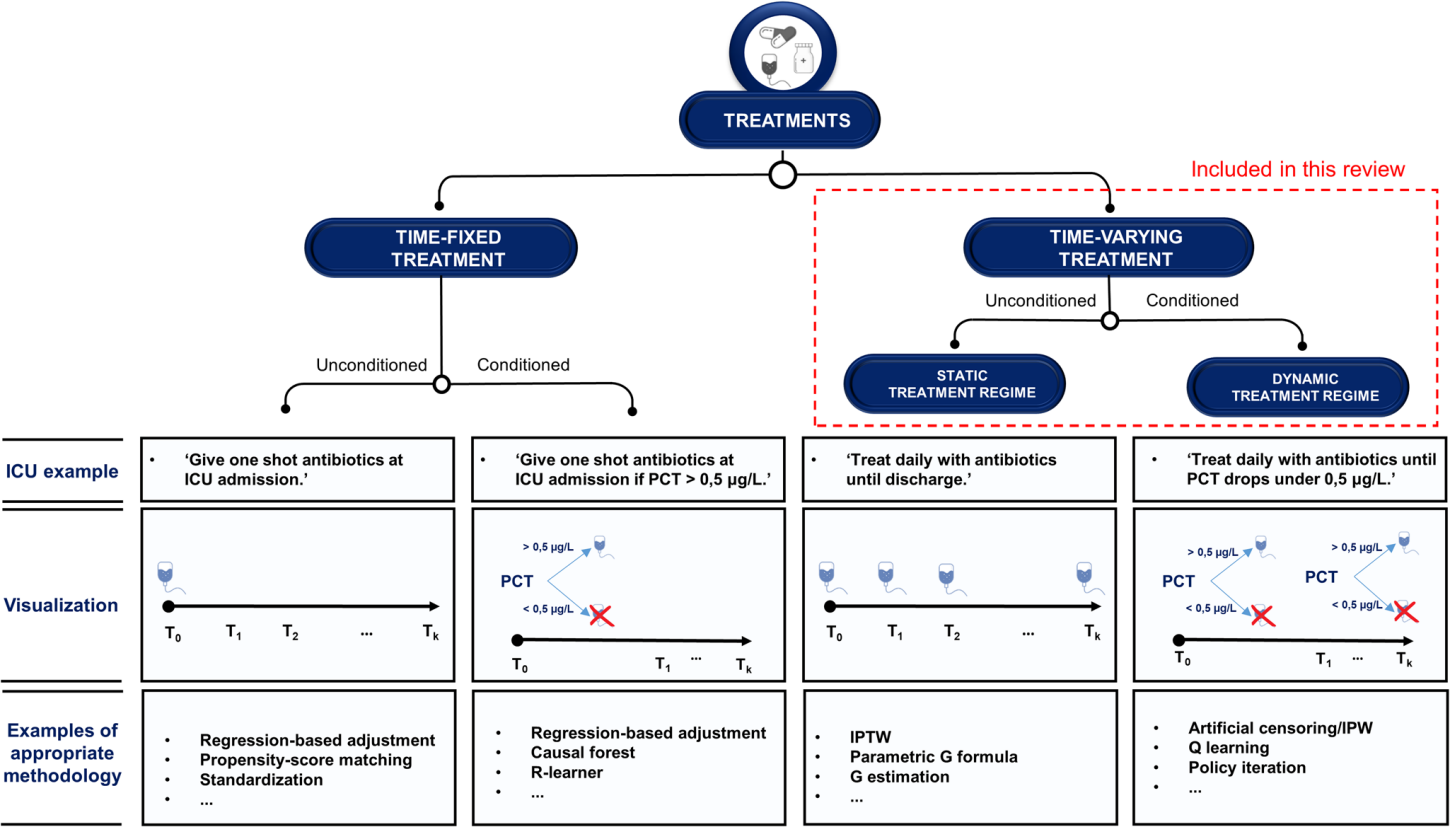
Taxonomy of treatment types. Treatments can be time-fixed or time-varying, and both these variants can be unconditioned, or conditioned on one or more (static and/or time-varying) patient characteristics, i.e., more ‘individualized’. Which methodology is appropriate to estimate causal effects of treatment using observational data, depends on the treatment type. The bottom row contains examples of some (not all) methodologies which could be used for the corresponding treatment type. Even when an appropriate method is used, satisfaction of the three causal assumptions (and hence, unbiased causal estimates) is not guaranteed. ICU intensive care unit, IPTW inverse-probability-of-treatment weighting, IPW inverse-probability weighting, PCT procalcitonin.

Many treatment choices in the intensive care unit (ICU) are made quickly, based on patient characteristics that are changing and monitored in real-time. Given this dynamic and data-rich environment, the ICU is pre-eminently a place where artificial intelligence (AI) holds the promise to aid clinical decision making^1–3^. So far, however, most AI models developed for the ICU remain within the prototyping phase^4,5^. One explanation for this may be that most models in the ICU are built for the task of prediction, i.e., mapping input data to (future) patient outcomes^6^. However, even a very accurate prediction of, for instance, sepsis^7^, does not tell a physician what to do in order to treat or prevent it. In other words, predictive AI is seldom actionable. More meaningful decision support in the ICU could be provided by models that assists clinicians in what to do (i.e., ‘actionable AI’^8,9^). To develop actionable AI, one needs to take into account cause and effect. Causal inference (CI) represents the task of estimating causal effects by comparing patient outcomes under multiple counterfactual treatments^6,10^. The most widely used method for CI is a randomized controlled trial (RCT). Through randomization (coupled with full compliance), the difference in outcome between treatment groups can be interpreted as a causal treatment effect. Because carrying out RCTs may be infeasible due to cost, time, and ethical constraints, observational studies are sometimes the only alternative. CI using observational data can be seen as an attempt to emulate the RCT that would have answered the question of interest (i.e., the ‘target trial’)^11^. With such an approach, however, treatment is not assigned randomly and extra adjustment for confounding is required. In the simple situation of a time-fixed (or ‘point’) treatment (Fig. 1, Box 1)^12,13^, this can be achieved by ‘standard methods’ like regression or propensity-score (PS) analyses^14^. However, ICU physicians are typically confronted with treatment decisions which occur at multiple time-points, i.e., time-varying treatments (Fig. 1, Box 1)^12,13^. Estimating the effect of time-varying treatments using observational data is often complicated by treatment-confounder feedback^15^, leading to ‘treatment-affected time-varying confounding’ (TTC, Box 1)^13,16,17^. Usage of standard methods in the presence of TTC leads to bias^18,19^. Inverse-probability-of-treatment weighting (IPTW), the parametric G formula and G estimation (collectively known as ‘G methods’, Box 1) were introduced by Robins^20^ to estimate causal effects in the presence of TTC, making them well-suited for CI in the ICU. Time-fixed treatments can be either unconditioned (e.g., ‘treat all patients at admission’) or conditioned on one or more covariates to make these more individualized (Fig. 1). Likewise, time-varying treatments can be either unconditioned, or tailored to more specific patient groups based on one or more (time-varying) covariates. We refer to these as static treatment regimes (STRs) and dynamic treatment regimes (DTRs), respectively (Fig. 1, Box 1)^12^. The latter type is most common in the ICU, as treatment choices are typically dynamically re-evaluated based on the evolving patient state. For example, rather than deciding upon admission to administer vasopressors daily, an ICU physician reconsiders giving this treatment throughout the ICU stay based on the patient’s response. Hence, the practical question of interest often requires a comparison of DTRs. Reinforcement learning (RL)^21^ is another class of methods which, like G methods, can be used to estimate optimal DTRs and have been increasingly applied to ICU data^22^. Partly due to the different language used to describe similar concepts (see Supplementary Table 1), studies applying G methods and RL may appear as completely separate disciplines. However, they show great similarities and can be used to build actionable AI models. The aim of this scoping review is to (1) outline how CI research is conducted concerning time-varying treatments in the ICU, (2) discuss quality of reporting, and (3) give recommendations to improve future research practice.

Box 1 Causal inference glossary

- **Time-fixed treatment**: a treatment that only occurs at the start of follow-up, or does not change over time.
- **Time-varying treatment**: any treatment that is not time-fixed. Time-varying treatments can be sub-divided in static and dynamic treatment regimes.
- **Static treatment regime (STR)**: a treatment regime that is not tailored to evolving patient characteristics.
- **Dynamic treatment regime (DTR)**: a treatment regime where the treatment decisions depend on changing patient characteristics and/or treatment history.
- **Treatment-affected time-varying confounding (TTC)**: time-varying confounding in which one or more time-varying confounders are affected by previous treatment.
- **G methods**: a class of methods proposed to adjust for TTC in the estimation of time-varying-treatment effects, including inverse-probability-of-treatment weighting, the parametric G formula, and G estimation.
- **Reinforcement learning (RL)**: a class of methods that deals with the problem of sequential decision making which returns an optimal treatment regime (or ‘policy’).
- **Off-policy evaluation (OPE)**: the task of estimating the value of a certain policy, using data from settings where patients were treated according to a different policy (i.e., observational data).
- **Causal assumptions:**
  • **Conditional exchangeability**: Exchangeability means equal risks in treated and untreated groups if patients in the untreated group were treated, and vice versa. Observational data often violates exchangeability due to confounding and/or selection bias, and, therefore, causal inference requires the assumption that all confounders are measured and adjusted for, to achieve exchangeability *conditional* on these confounders.
  • **Positivity**: To estimate a treatment’s causal effect, one must compare treated and untreated patient data. This requires having both treated and untreated patients in all subgroups (or ‘strata’) defined by different confounder values. In other words, treatment and non-treatment should occur in all confounder strata with some *positive* probability.
  • **Consistency**: Consistency assumes that the outcome for a given treatment will be the same, irrespective of the way treatments are ‘assigned’.

## Results

**Fig. 2:**
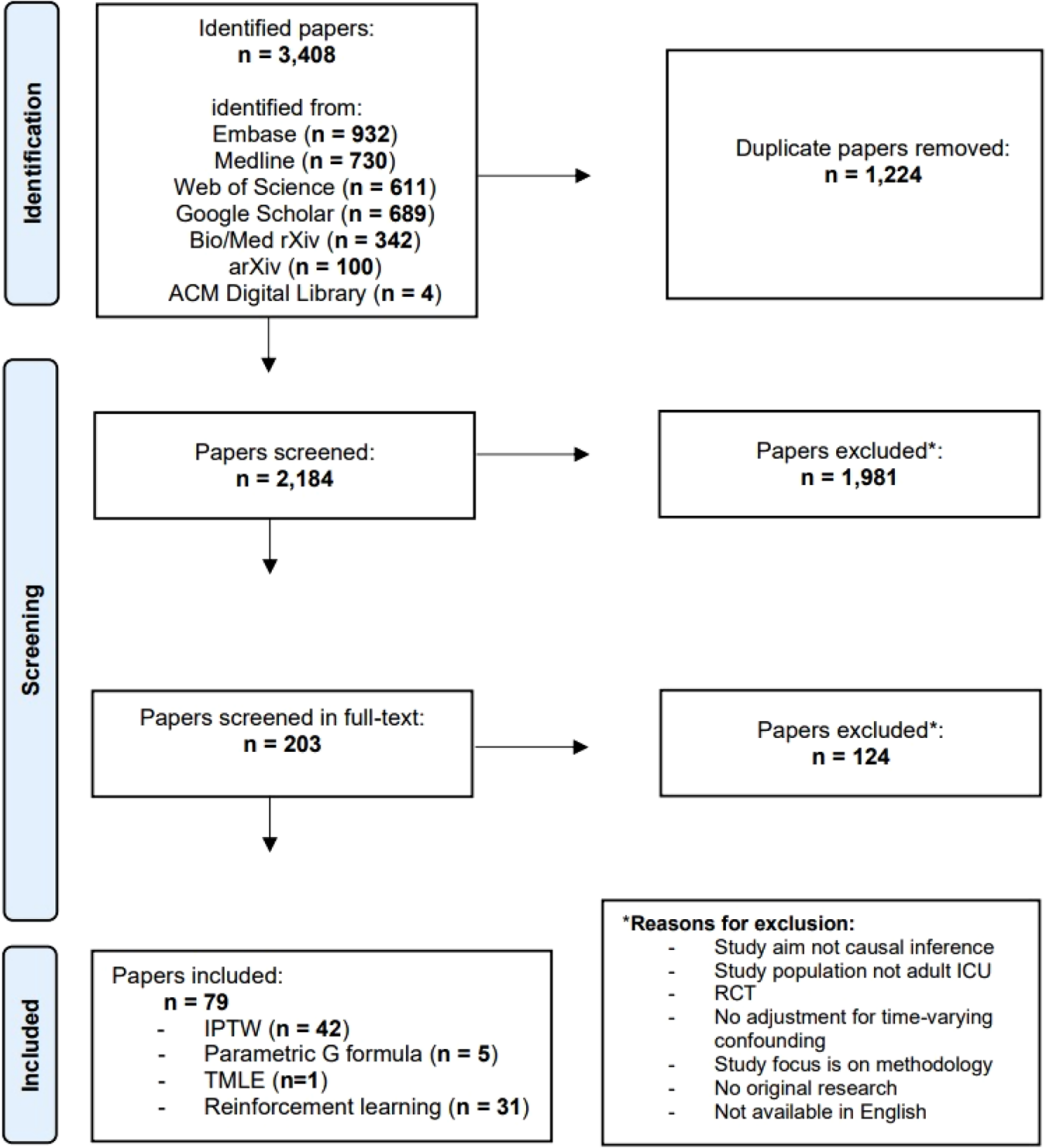
Flowchart of study selection. This flow diagram displays the screening strategy for the inclusion of studies in this scoping review.

Through our search, we discovered 2184 articles that were unique. During the independent screening of titles and abstracts, we achieved a high agreement rate of 93%. Following the resolution of any disagreements, we excluded 1981 studies. Moving forward, we conducted independent full-text screening for the remaining 203 articles, resulting in an agreement rate of 86%. Once a consensus on eligibility was reached, we included 79 studies in the review (Fig. 2). The articles were published between 2005 and 2023, with a steadily growing number of articles per year starting around 2010 (Supplementary Fig. 1a). A reference list of all included studies and the list with collected items per study can be found in the Supplementary References and supplementary Table 2, respectively. Most studies applied G methods (*n* = 48, 61%), of which 42 (53%) used IPTW, five (6%) used the parametric G formula. One (1%) study^23^ used targeted minimum loss-based estimation (TMLE), a method which combines elements of IPTW and the parametric G formula^24^. 31 (39%) studies used RL methods (Table 1). The three most frequently studied treatment categories were anti-inflammatory drugs (*n* = 9, 11%), hospital-acquired complications (*n* = 8, 10%), and sedatives and analgesics (*n* = 8, 10%). Most studies (*n* = 46, 58%) considered mortality (at varying follow-up times) as the primary outcome. Thirty-six studies (46%) included data from at least two different ICUs. Studies that used RL generally included more patients than studies that used G methods, with a median of 9782 patients (IQR 5022–17,898) versus 1498 (IQR 606–3407) and relied more often on open-source ICU databases (74% vs 23%). In total, 34 (43%) of the studies used one or more open-source ICU database, among which the Medical Information Mart for Intensive Care (MIMIC)-III database^25^ was the most frequently used (*n* = 24, 30%). In contrast to RL studies (which inherently deal with DTRs), only eight^26–33^ of the 48 studies (17%) that used G methods considered DTRs and seven of these were published in or after 2020 (Supplementary Fig. 1b).

**Table 1:** Characteristics of the included studies grouped by used causal inference method.

	IPTW (*N* = 42) No (%)	Parametric G formula (*N* = 5) No (%)	TMLE (*N* = 1) No (%)	RL (*N* = 31) No (%)	All (*N* = 79) No (%)
Exposure of interest (categorized)					
Vasopressors & intra-venous fluids	0 (0)	0 (0)	0 (0)	10 (32)	10 (13)
Anti-inflammatory drugs	7 (17)	0 (0)	0 (0)	2 (6)	9 (11)
Hospital-acquired complications	8 (19)	0 (0)	0 (0)	0 (0)	8 (10)
Sedatives & analgesics	1 (2)	0 (0)	0 (0)	6 (19)	7 (9)
Antimicrobials	4 (10)	0 (0)	0 (0)	0 (0)	4 (5)
Mechanical ventilation	1 (2)	2 (40)	0 (0)	3 (10)	6 (8)
Anticoagulants	1 (2)	0 (0)	0 (0)	2 (6)	3 (4)
Diuretics	3 (7)	0 (0)	0 (0)	0 (0)	3 (4)
Renal replacement therapy	3 (7)	0 (0)	0 (0)	0 (0)	3 (4)
Sodium bicarbonate	3 (7)	0 (0)	0 (0)	0 (0)	3 (4)
Blood transfusion	2 (5)	0 (0)	0 (0)	1 (3)	3 (4)
Other	9 (21)	3 (60)	1 (100)	7 (23)	20 (25)
Primary outcome (categorized)					
Mortality	32 (76)	2 (40)	1 (100)	11 (35)	46 (58)
Combined	0 (0)	0 (0)	0 (0)	13 (42)	13 (16)
Maintenance of clinical target value	0 (0)	0 (0)	0 (0)	6 (19)	6 (8)
Hospital-acquired complications	1 (2)	2 (40)	0 (0)	0 (0)	3 (4)
Need for mechanical ventilation	2 (5)	0 (0)	0 (0)	0 (0)	2 (3)
Other	7 (17)	1 (20)	0 (0)	1 (3)	9 (11)
Number of included ICUs^a^					
1	16 (38)	2 (40)	0 (0)	20 (77)	38 (51)
2–4	5 (12)	1 (20)	1 (100)	4 (15)	11 (15)
5–10	3 (7)	1 (20)	0 (0)	0 (0)	4 (5)
11–20	4 (10)	1 (20)	0 (0)	0 (0)	5 (7)
21–100	5 (12)	0 (0)	0 (0)	0 (0)	5 (7)
>100	9 (21)	0 (0)	0 (0)	2 (8)	11 (15)
Utilized open source databases					
MIMIC-II	0 (0)	0 (0)	0 (0)	1 (3)	1 (1)
MIMIC-III	6 (14)	0 (0)	0 (0)	18 (58)	24 (30)
MIMIC-IV	3 (7)	1 (20)	0 (0)	3 (10)	7 (9)
eICU	2 (5)	0 (0)	0 (0)	2 (6)	4 (5)
AmsterdamUMCdb	0 (0)	1 (20)	0 (0)	2 (6)	3 (4)
Study size (*n* patients)^a^					
0–100	2 (5)	0 (0)	0 (0)	0 (0)	2 (3)
100–500	7 (17)	1 (20)	0 (0)	0 (0)	8 (11)
501–1000	9 (21)	0 (0)	1 (100)	1 (4)	11 (15)
1001–5000	16 (38)	3 (60)	0 (0)	6 (23)	25 (34)
>5000	8 (19)	1 (20)	0 (0)	19 (73)	28 (38)
Type of exposure regime					
Static	36 (86)	3 (60)	1 (100)	0 (0)	40 (51)
Dynamic	6 (14)	2 (40)	0 (0)	31 (100)	39 (49)

*IPTW* inverse probability of treatment weighting, *TMLE* targeted minimum loss-based estimation, *RL* reinforcement learning.

^a^For the number of included ICUs and study size, the studies that used simulated patient data (*n* = 5) are not taken into account and therefore, the number of RL and all studies add up to 26 and 74, respectively.

### Method-specific items

Among the studies that used IPTW (*n* = 43), eighteen applied stabilized weights, one applied weight truncation, and ten studies applied both weight stabilization and truncation. The remaining studies did not apply weight stabilization or truncation. Among studies that applied RL on real (i.e., not simulated) patient data (*n* = 26), fourteen studies used either an importance-sampling based^34^, model-based^35,36^, a doubly robust off-policy evaluation (OPE, Box 1) method^37^, or a combination of these. Thirteen studies used the so-called ‘U-curve method’^38^ (Box 1) and for nine of these, this was the only reported OPE method. Two studies did not report any evaluation of the optimized DTR (Supplementary Fig. 2).

### Quality of reporting

A total of 1183 unique quality of reporting (QOR) items were assessed independently, encompassing the eight, eleven, and ten target trial subcomponents for the IPTW/TMLE, parametric G formula, and RL papers, respectively, along with six distinct items for the causal assumptions in all included studies. Initially, the reviewers agreed on 1012 items (86%), and any remaining disagreements were resolved through discussion until consensus was reached. As each subcomponent of the analysis plan component required for IPTW studies also applies to TMLE, we used the same subcomponents to judge the analysis plan component of the study that used TMLE^23^.

#### Target trial components

**Fig. 3:**
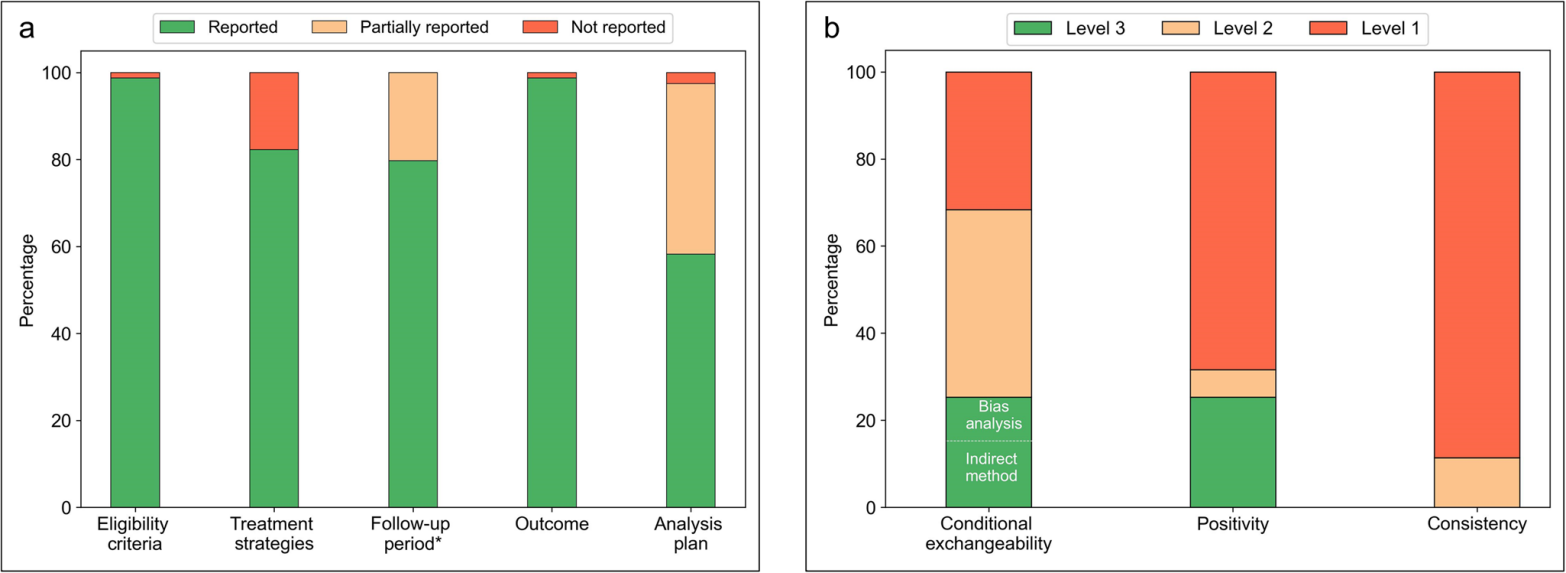
Quality of Reporting summary plots. **a** Reporting quality of the target trial components. *For the follow-up component, the studies that used simulated patient data (*n* = 5) are not taken into account. **b** Reporting quality of causal assumptions (level 1 = assumption not mentioned, level 2 = assumption mentioned, level 3 = attempt to check for potential violations of the assumption reported).

Both the ‘eligibility criteria’ and ‘outcome’ components were reported in 78 (99%) of the studies (Fig. 3a). We scored the ’treatment strategies’, ‘follow-up period’ and ‘analysis plan’ components as partially or not reported in respectively 14 (18%), 16 (20%) and 33 (42%) of the studies. All five target trial components were fully reported in only 30 (38%) studies. The reporting of the target trial components grouped by used CI method are summarized in Supplementary Figs. 3–5 and tabulated for each individual study in Supplementary Tables 3–5.

#### Causal assumptions

The conditional exchangeability assumption remained unmentioned in 25 (32%), was mentioned in 34 (43%), and an attempt to check for potential violations was reported in 20 studies (25%, Fig. 3b). Among the studies that reported a check for potential violations, eight studies^31,33,39–44^ performed a bias analysis. The positivity assumption remained unmentioned in 54 (68%), was mentioned in five (6%), and a check for potential violations was reported in 20 (25%) of the studies. The consistency assumption was mentioned in nine (11%) of the studies. All three assumptions were mentioned (or a check for potential violations was reported) in only seven (9%) studies (Supplementary Table 6). The reporting of assumptions grouped by CI method used are summarized in Supplementary Figs. 6–8 and individual results for all studies are tabulated in Supplementary Table 6. In general, the causal assumptions remained unmentioned more often in studies that applied RL, compared to those which applied G methods (Supplementary Figs. 6–8). All studies that reported a check for potential violations of the conditional exchangeability assumption also mentioned this assumption, whereas for the positivity assumption, eleven out of 20 studies that reported a check for potential violations did not explicitly mention positivity (Supplementary Table 6).

### Adjusting for treatment-affected time-varying confounding

**Fig. 4:**
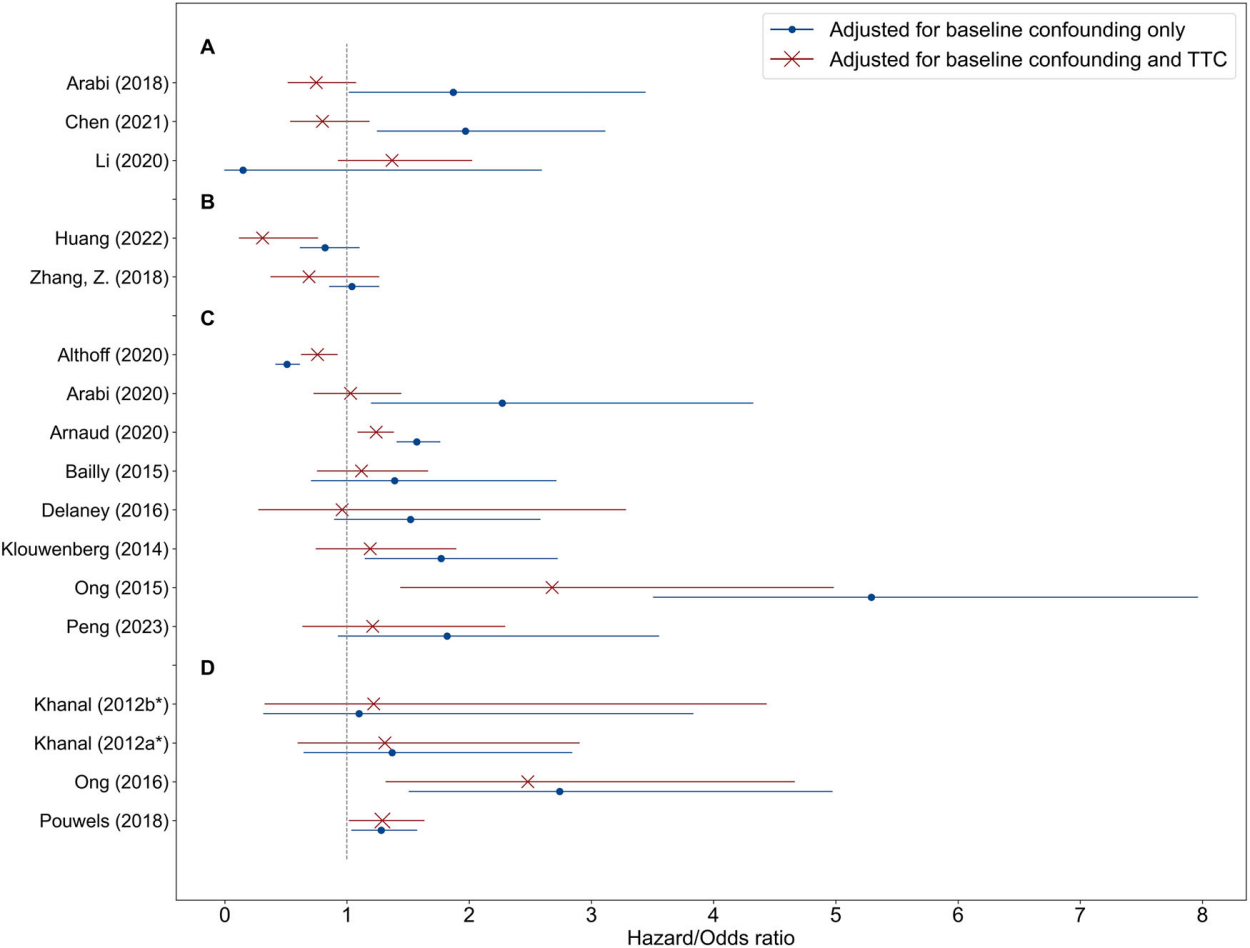
Influence of adjusting for treatment-affected time-varying confounding. Treatment effect estimates adjusted only for baseline confounding versus adjusted for baseline and treatment-affected time-varying confounding (TTC) reported by sixteen of the included studies. Treatment effects were reported in terms of odds or hazard ratios (including the reported 95% CIs). In three studies (**A**), the point estimates moved to the opposite direction, in two (**B**) and eight (**C**) studies, the estimates moved away from and towards the null hypothesis, respectively. In three studies (**D**), there was a marginal difference in point estimates. Pouwels et al. (2020) estimated treatment effect on length-of-stay (in terms of days) by adjusting for baseline confounding and by adjusting for baseline confounding and TTC, and found a marginal difference in point estimates. *Khanal et al. (2012) compared prolonged intermittent renal replacement therapy with *two different alternative* treatments.

Seventeen studies (22%) estimated the treatment effect by adjusting for baseline confounding and by adjusting for baseline confounding and TTC. For most of these studies, the point estimates of the treatment effects varied substantially after adjusting for both baseline and TTC, moving the effect estimate towards or away from the null hypothesis, or even leading to opposite effect estimates (Fig. 4).

## Discussion

Our scoping review of 79 published studies revealed a diverse range of treatments being investigated. Despite the fact that most treatments of interest in the ICU setting are DTRs, we observed a dominant emphasis on STRs among studies that used G methods. Many studies had inadequate reporting of the components of target trials, with the ‘treatment strategies’, ‘follow-up’ and ‘analysis plan’ components being incompletely reported most frequently. The causal assumptions were frequently not specified, especially in studies utilizing RL methods, indicating a potential lack of awareness in this research field of the importance of these assumptions. The upcoming ‘Prediction of Counterfactuals Guideline’ (PRECOG)^45^ aims to provide guidance on the reporting of causal assumptions and model evaluation in CI studies using observational data, and we anticipate that its adoption may yield substantive enhancements in the quality of reporting in forthcoming CI studies utilizing observational ICU data. We decided not to employ the ROBINS-I tool^46^ for assessing bias in observational studies in our evaluation. We made this choice for two main reasons. Firstly, utilizing ROBINS-I would require verifying specific causal assumptions, which, in turn, would demand expert knowledge of the treatment-outcome relationships studied in each of the included articles. Given the extensive scope of our review and the fact that many studies did not explicitly address these causal assumptions, we found this task to be beyond the intended scope of our review. Consequently, we opted to evaluate studies based on their acknowledgment of these assumptions and their reported attempts to validate them. Secondly, when conducting causal inference with observational data, the potential for bias arises not only from the absence of randomization but also from potential biases stemming from incorrect study design choices^47^. Rather than assessing these design choices directly, we chose to evaluate studies based on the clarity and completeness of their reporting regarding these choices. To structure this evaluation, we used the target trial framework^11^, which is widely recognized and used as a conceptual benchmark to describe study design choices in causal inference studies using observational data across various medical domains^48–52^. G methods and RL methods are often perceived as separate disciplines, but show great similarities^53^. For example, Q-learning^54^ (an RL method, used by many of the included studies^55–59^) is very similar -and under certain conditions even algebraically equivalent- to G estimation (a G method)^60^. An important difference is that G methods are used for modeling both STRs and DTRs, while RL methods typically deal with DTRs. As both G methods and RL methods perform the same CI task (i.e., finding optimal treatment regimes), both rely on the same, strong causal assumptions, which should be acknowledged. While the consistency assumption is often plausible for treatments in the ICU, violations of the conditional exchangeability and positivity assumption are more likely and should be examined. Prior to examining violations of the causal assumptions, one needs a research question that is truly of interest in the ICU, a clear description of study design choices (e.g., using the target trial framework^11^), and usage of a CI method that is appropriate for the type of studied treatment. The results of our scoping review have led to five recommendations that build on widely accepted views and concepts in the field of CI to improve future research and move towards actionable AI in the ICU (Box 2).

Box 2 Summary of recommendations for future research

- **Ask the right research question**
  When developing a model for causal inference, consider clinically relevant treatments. In the ICU, treatment decisions typically occur at multiple time point during admission (i.e., time-varying treatments) and often depend on the patient’s response to treatment (i.e., dynamic treatment regimes).
- **Describe the question as a target trial emulation**
  It is useful to imagine a randomized trial that would have answered the research question (i.e., the target trial). Even if performing the target trial is not feasible, describing its components using the target trial framework helps to identify flaws in the relevance of a research question and correctness of the analysis.
- **Use methods that suit the research question**
  Standard methods, like propensity-score analyses, are easy to implement and suffice for time-fixed treatments, but lead to biased estimates when used to adjust for treatment-affected time-varying confounding (TTC). Research questions concerning time-varying treatments are often complicated by TTC, and, therefore, require methods that can appropriately adjust for this.
- **Mind the conditional exchangeability assumption**
  Causal inference is not possible based on data only, and incorporation of expert knowledge is key to think about the causal structure between the treatment and outcome of interest. Representing this expert knowledge in causal diagrams is useful to visualize potential sources of bias. A bias analysis can be helpful to quantify how much bias unmeasured confounders could produce.
- **Mind the positivity assumption**
  This assumption is verifiable, but this is rather complex for time-varying treatments and violations are expected given the dynamic nature of the ICU. Violations could be minimized by increasing the sample size (e.g., by more usage of open-source ICU databases) and by studying treatment regimes that are (more) similar to those observed in the data. Examination of estimated inverse-probability-of-treatment weights is useful to detect (but not rule out) positivity violations.

### Ask the right research question

Treatments of interest in the ICU are typically DTRs and, therefore, this type of treatments is expected to be the focus of CI research in the ICU. However, despite an upward trend in recent years (Supplementary Fig. 1b), still 83% of the studies that used G methods studied STRs. To illustrate that many of these studies are considering research questions that are not truly of interest in the ICU, we will explore some examples. Zhang and colleagues divided patients into two groups according to whether they received diuretics within the first two days of ICU admission or not^61^. Thus, the emulated target trial answers the question whether or not to administer diuretics at the start of ICU admission. However, we argue that the question an ICU physician is really interested in is when to administer diuretics throughout the whole ICU stay, taking into account changing patient characteristics such as fluid balance (especially at later ICU stages). In addition, many of the included studies emulated target trials comparing ‘giving treatment sometime during follow-up’ versus ‘never giving treatment’. For example, Bailly and colleagues studied the effect of systemic antifungal therapy, comparing a treated group (those who received antifungals during their ICU stay) with an untreated group (those who never received antifungals)^62^. As giving treatment ‘sometime during follow-up’ can be done in many ways, the estimated treatment effect is ill-defined and typically not truly of interest. In other words, both studies by Zhang^61^ and Bailly^62^ serve as examples of emulated RCTs that would never be conducted in the ICU. Conversely, Morzywołek and colleagues^27^ emulated a target trial that reflects a very relevant research question, i.e., what is the optimal moment to start renal replacement therapy in acute kidney injury? They identified an optimal DTR based on a combination of biomarkers lowered mortality without increasing the number of RRTs, offering valuable insights for planning a future confirmatory RCT. Wang and colleagues investigated the effectiveness of low tidal volume ventilation^32^, a DTR investigated in one of the most influential RCTs in the field of intensive care medicine^63^. This trial faced criticism (among other concerns) due to high non-adherence rates. Consequently, the target trial these authors emulated reflected the highly relevant question: what would the treatment effect have been under full compliance?

### Describe the question as a target trial emulation

It can be beneficial to conceptualize a randomized trial that could have addressed the research query (i.e., the target trial). Even in cases where executing the exact target trial is unfeasible, outlining its constituent elements within the target trial framework^11^ aids in recognizing shortcomings in the appropriateness of the research question and accuracy of the analysis. Almost one fifth of the included studies lacked a clear description of the ‘treatment strategies’ component of the target trial, that is, which treatment regimes are compared in the target trial. For example, Arabi and colleagues^64^ used IPTW to study the effect of corticosteroid therapy for ICU patients with Middle East Respiratory Syndrome. However, it remains unclear which treatment regimes (e.g., ‘treat daily with corticosteroids’) are being compared. Moreover, more than one third of the included studies lacked a complete description of the ‘analysis plan’ component and therefore, are not reproducible. We advocate detailed description which allows reproduction of the used methodology, ideally accompanied with code and (example) data.

### Use methods that suit the research question

Many studies were not included in this review because they modeled time-fixed treatments. As time-fixed treatments in the ICU are rare, we hypothesize that in many of these studies, the implicit treatment of interest is time-varying. Research questions concerning time-varying treatments may be reformulated into simplified, time-fixed versions, because standard methods are easier to implement or high-quality, longitudinal data is unavailable. One may argue that, if the bias introduced by TTC^18,19^ is negligible, standard methods suffice for CI in time-varying treatments as well. However, empirical results from studies included in this review suggest that adjusting for TTC can lead to substantial differences in effect estimates and sometimes even to opposite conclusions (Fig. 4). Hence, it is possible that many of the excluded studies that implicitly studied time-varying treatments but modeled these as if they are time-fixed, published biased effect estimates. We advocate adjustment for TTC in any CI study where the treatment of interest is time-varying. Modeling DTRs is slightly more complex than STRs (which may be a reason for the focus on STRs among the included studies) and therefore requires different approaches. Various methods exist to find optimal DTRs, either from a set of pre-specified regimes or directly from data (for an overview, we refer to the book by Chakraborty and Moodie^65^). Among the included studies in this review, for example, Shahn and colleagues^28^ used ‘artificial censoring/IPW’^65–67^ to estimate the optimal fluid-limiting treatment regime for sepsis patients among a pre-specified set of DTRs (i.e., ‘fluid caps’). RL methods and G estimation can be used to approximate optimal DTRs without a pre-specified set of regimes. In RL studies, finding the optimal treatment regime (typically referred to as the optimal ‘policy’, see Supplementary Table 1) is typically followed by a validation step to quantify the value of the optimized regime (i.e., OPE, Box 1). The ‘U-curve method’^38^, a frequently used OPE method among the included RL studies in this review (Supplementary Fig. 2), is based on associating the difference between the (observed) clinician’s treatment regime and the (estimated) optimal treatment regime with patient outcome. As it completely ignores the potential effect of confounders, we recommend avoiding this method.

### Mind the conditional exchangeability assumption

Conditional exchangeability is never guaranteed using observational data as the absence of unmeasured confounders is not verifiable in the data. To think about residual confounding or selection bias, incorporation of subject-matter expertise is key. Directed acyclic graphs (DAGs)^68^ provide a simple and lucid approach for researchers dealing with observational data to showcase this expert knowledge, theories, and suppositions regarding the causal relationships among variables. For practical guidance on effective utilization of DAGs we refer to the work of Tennant and colleagues^69^. There are different approaches to quantify how potential violations of the conditional exchangeability would affect the study results^70^. Indirect approaches consider, for instance, the effect of adding additional confounders^11^. A ‘bias analysis’ (or sensitivity analysis)^71^ examines the characteristics of potential unmeasured confounders and can be useful to quantify how much bias it would produce as a function of those characteristics.

### Mind the positivity assumption

The positivity assumption -on the contrary- is verifiable, although this is rather complex for time-varying treatments^72^ and, given its dynamic nature, violations are expected in the ICU setting. The intuition for this assumption is that one can only study a treatment regime using data of patients who have received treatment that conform to this regime. The number of patient treatment histories that match the treatment regime of interest (i.e., the ‘effective sample size’^73^) shrinks with the number of treatment decisions in the patient’s history (which tends to be high in the ICU). For example, Gottesman and colleagues^38^ applied RL to a dataset of 3855 patients to find an optimal treatment regime for sepsis, but the effective sample size for this regime was only a few dozen. A small effective sample size makes positivity222222 violations likely and leads to high uncertainties in estimated treatment effects. A straight-forward opportunity to tackle this challenge is increasing the sample size. Therefore, we advocate more usage (if appropriate) of the four currently available open-source ICU databases^74^. However, increasing the sample size does not guarantee increasing the *effective* sample size, as the patients in the extra dataset may not be treated according to the regime of interest. Hence, another opportunity to increase the effective sample size is to minimize the mismatches between the treatment regime(s) of interest and those observed in the data. Studies employing G methods may simply accomplish this by avoiding the modeling of treatment regimes which differ greatly from the treatment protocol in place. In contrast, studies utilizing RL typically do not pre-specify treatment regimes (as they optimize them through learning agents) and consequently, avoiding specific treatment regimes (or policies) is more challenging. In the RL literature, various approaches have emerged to address this issue, resulting in policies more closely aligned with physician practices^75^. These approaches can be categorized into ‘policy constraint’ and ‘uncertainty based’ methods, with the latter exemplified by the application of Conservative-Q Learning in two of the included RL studies^76–78^. Detection (but not ruling out) of violations of the positivity assumption can be facilitated through examination of the distribution of estimated (stabilized) inverse-probability-of-treatment (IPT) weights^72^, which was done in 20 of the 42 studies that utilized IPTW (Supplementary Table 6). This examination is recommended not only for IPTW but also for studies utilizing other CI methods. In studies using RL methods that employ importance-sampling^34^ (which is closely related to IPTW^53^) for OPE, analogous to the examination of IPT weights, it is recommended to examine the distribution of the importance weights^38^. In studies using IPTW, weight stabilization and truncation can be used to limit high uncertainties in the effect estimates. Weight stabilization can improve the precision of effect estimates without the introduction of bias. However, a model based on stabilized weights results in a slightly different effect estimate compared to non-stabilized weights^79^ and, therefore, should be interpreted carefully. Weight truncation also improves precision, but at the expense of bias. Examination of the influence of the introduced bias by checking the change of the effect estimates under progressive truncation of IPT weights is recommended^80^.

This review stresses the importance of causality for actionable AI and provides a contemporary overview of CI research in the ICU literature. We describe shortcomings of the identified studies in terms of reporting and, furthermore, provide handles for improving future CI research. These recommendations are not limited to the ICU but apply to medical CI research as a whole. Unlike other reviews on time-varying medical treatments^22,81,82^, we did not limit our focus to either G methods or RL, but rather acknowledge that both these method classes can be used to perform CI tasks and therefore, hold the promise to bring actionable AI to the bedside.

Our review has limitations. First, whereas efforts were made to ensure that the literature search was comprehensive, we could have missed studies for different reasons. Some research might have employed unconventional terminology to delineate their chosen CI method or utilized a CI approach that fell outside the scope of our search strategy. For example, dynamic weighted ordinary least squares (dWOLS)^83,84^ is a relatively new method which has been used to model DTRs in the ICU setting in several studies^85,86^. This method benefits from properties of both Q-learning (an RL method) and G-estimation (a G method) and may be an interesting direction for future research. Also, digital twin technology builds on causal inference and has been applied in the ICU setting^87^. Second, items that were not collected could be of interest for future investigation. For example, we did not differentiate RL further into specific RL methods.

Towards actionable AI in the ICU, we concur with the guidance of editors of critical care journals^88,89^ to change the focus of observational research in the ICU from prediction to causal inference. To unlock this potential in a trustworthy and responsible manner, we advocate development of models for CI focusing on clinically relevant treatments, using a description of the research question as a target trial emulation, choosing appropriate CI methods given the treatment of interest and acknowledging (and ideally examining potential violation of) the causal assumptions.

## Methods

The study protocol was registered in the online PROSPERO database (CRD42022324014)^90^. The filled-in PRISMA Extension for Scoping Reviews (PRISMAScR) checklist^91^ can be found in Supplementary Table 7.

### Search strategy

Candidate articles were identified through a comprehensive search in Embase, MEDLINE ALL, Web of Science Core Collection, Google Scholar, medRxiv, bioRxiv, arXiv and ACM Digital Library up to March 2023, with no start date. We developed a search strategy that could be modified for each database (Supplementary Table 8). Search terms included relevant controlled vocabulary terms and free text variations for CI, G methods, or common RL methods, combined with ICU related terms.

### Eligibility criteria

We included any primary research article, conference proceedings or pre-print papers that present models for the task of CI concerning time-varying treatments in patients admitted to the adult ICU. Articles were not eligible if it modeled a time-fixed treatment, utilized data from an RCT (unless the treatment of interest was not the randomized treatment), it focused on the introduction of new methodology, or was an abstract-only, review, opinion, or survey. Duplicates and articles not written in English were also excluded.

### Study selection

Duplicate removal and eligibility screening were performed using EndNote 20 (Clarivate Analytics, Philadelphia, PA, USA). We used a two-stage approach for screening: first by title and abstract and then by full article text. Two reviewers (JS and WK) independently screened the titles and abstracts. The two reviewers then independently performed full-text screening on all articles. Conflicts regarding the eligibility of studies during the screening process were resolved by consensus in regular sessions between the two reviewers.

### Data extraction

Data were extracted by JS and confirmed by WK. The items for extraction were based on the STROBE (STrengthening the Reporting of OBservational studies in Epidemiology) checklist^92^, supplemented by method-specific items. We extracted the following items from all included studies: details of study variables (i.e., studied treatment and primary outcome), the number of included ICUs, usage of open-source database(s), number of participants included, studied treatment type (Fig. 1), and used CI method. In addition, we extracted the following method-specific items: the usage of methods to reduce extreme weights (i.e., weight stabilization^93^ and truncation^80^) for studies using IPTW and the off-policy evaluation^94^ (OPE, Box 1) method used for studies using RL. Finally, if a study estimated the treatment effect both by adjusting for baseline confounding and by adjusting for baseline confounding and TTC, we also collected these different estimates.

### Quality of reporting

To assess the quality of reporting (QOR) of the included studies, two reviewers (JS and WK) independently judged the reporting of the components of the target trial framework^11^ and the causal assumptions (Box 1). Conflicts regarding the QOR assessment were resolved by consensus in regular sessions between the two reviewers.

### Target trial components

The ‘target trial framework’, introduced by Hernán and Robins^11^, consists of seven main components. We judged the reporting of five of these: eligibility criteria, treatment strategies, follow-up period, outcome and analysis plan (Table 2). We scored the analysis plan component as ‘reported’ if one could reproduce the modeling given the required data. For studies using RL, we judged the ‘treatment strategies’ and ‘outcome’ components as ‘reported’ if the definitions of the used action space and reward were reported, respectively. We split the follow-up period component into three subcomponents: time-zero (or ‘index date’), end of follow-up, and time resolution (i.e., the time steps in which the treatment level is considered the same)^95^. We split the analysis plan component into specific subcomponents depending on the CI method used (Supplementary Table 9). We scored the target trial components that are split in subcomponents as ‘reported, ‘partially reported’ and ‘not reported’ if all, some, or none of the subcomponents were reported, respectively. Leading questions used to judge whether each target trial subcomponent was reported can be found in Supplementary Table 9. We did not evaluate QOR for the components of ‘assignment procedures’ and ‘causal contrast of interest’, as no variability among the studies was expected. This is due to the fact that (except during clinical trials) ICU physicians typically have full knowledge of the treatments being administered to patients, resulting in an unblinded assignment of the modeled treatment. Additionally, the primary objective in CI studies using observational data is typically to compare the effect of actual treatment adherence (per-protocol effect), rather than the effect of being assigned to a specific treatment regime at baseline (intention-to-treat effect).

**Table 2:** Components of the target trial framework included in the reporting quality assessment with examples for the ICU setting.

Component	Subcomponent	Included for QOR assessment	ICU example
Eligibility criteria	–	✓	Individuals aged 65 years or older admitted to the ICU meeting Sepsis-3 criteria upon admission.
Treatment strategies	–	✓	1. Liberal fluid therapy (administration of intravenous fluid boluses during the first several hours of treatment) 2. Restrictive fluid therapy (Intravenous fluid boluses during ICU stay in only in case of severe hypoperfusion)
Assignment procedures	–		Unblinded, random assignment to one of the treatment strategies.
Follow-up period	Start of follow-up	✓	Time of ICU admission.
	End of follow-up	✓	Death, ICU discharge, or loss to follow-up, whichever occurs first.
	Time resolution	✓	Hourly weights were applied to adjust for the impact of time-varying confounders on the hourly risk of adhering to one of the two treatment strategies.
Outcome	–	✓	All- cause mortality.
Causal contrasts of interest	–		Per-protocol effect.
Analysis plan^a^		✓	Per-protocol effect will be estimated adjusting for pre- and postbaseline confounders by a marginal structural model using IPTW.^b^

^a^The analysis plan component is subdivided in a specific set of subcomponents depending on the modeling strategy used, these are summarized in Supplementary Table 9.

^b^This description would not be considered as sufficient reporting of the analysis plan component, but simply serves as an example.

### Causal assumptions

The task of CI relies on strong assumptions, including conditional exchangeability, positivity, and consistency (referred to collectively as ‘causal assumptions’, Box 1). Violations of these assumptions result in biased estimates and therefore it is crucial to acknowledge them and, if possible, assess potential violations. A study’s QOR regarding causal assumptions was scored using three levels of increasingly good reporting quality: (1) no mention of the assumption, (2) mention of the assumption, and (3) attempted examination of potential assumption violations. For the conditional exchangeability assumption, two types of attempts were differentiated: ‘indirect approaches’^11^ and ‘bias analyses’^71^. The examination of the distribution of the (stabilized) IPT weights was considered as a method to assess potential positivity assumption violations. As there are currently no approaches to check for consistency assumption violations, merely mentioning the consistency assumption (level 2) was considered the highest level of reporting quality.

### Evidence synthesis

We tabulated extracted study items for each study individually and grouped by CI method used. QOR results concerning the target trial components and the causal assumptions are summarized as percentages using bar charts, and results of the QOR assessment for each study individually were presented in a long format table. For the reporting of the target trial components, we made separate tables for each group of studies that used a specific CI method. The collected treatment effect estimates reported by studies that estimated the treatment effect both by adjusting for baseline confounding and by adjusting for baseline and TTC, were plotted as point estimates and corresponding 95% confidence intervals.

### Supplementary information

Supplementary Information

## Data Availability

The authors declare that all data supporting the findings of this study are available within the paper and its Supplementary Information.
