# Supplementary material for: Causal inference using observational intensive care unit data: a scoping review and recommendations for future practice
Source: NPJ Digit Med. 2023 Nov 27;6:221. doi: 10.1038/s41746-023-00961-1 (PMC10682453; doi:10.1038/s41746-023-00961-1)
Supplement: Supplementary file 1 — Supplementary Information [file 41746_2023_961_MOESM1_ESM.pdf]

Supplementary Information

Causal inference using observational intensive care unit data: a scoping review and recommendations for future practice

J. M. Smit[1,2], J. H. Krijthe[2], W.M.R. Kant[3], J.A. Labrecque[4], M. Komorowski[5,6], D.A.M.P.J. Gommers[1], J. van Bommel[1], M. J. T. Reinders[2], M.E. van Genderen[1]

[1] Department of Intensive Care, Erasmus University Medical Center, Rotterdam, The Netherlands
[2] Bioinformatics Pattern Recognition & group, EEMCS, Delft University of Technology, Delft, The Netherlands
[3] Data Science group, Institute for Computing and Information Sciences, Radboud University, Nijmegen, The Netherlands
[4] Department of Epidemiology, Erasmus Medical Center, Rotterdam, Netherlands
[5] Department of Surgery and Cancer, Faculty of Medicine, Imperial College London, London, UK
[6] Intensive Care Unit, Charing Cross Hospital, Imperial College Healthcare NHS Trust, London, UK

Supplementary Tables

Supplementary Table 1: Commonly used terms (not synonyms) to describe similar concepts typically used in research using G methods and reinforcement learning methods.

**G methods**	**Reinforcement learning**	**Other commonly used terms**
Treatment	Action	Exposure, intervention
Outcome	Reward	
(Treatment) regime	Policy	strategy, regimen, decision rule, joint exposures, sustained strategy, plan, protocol
Structural causal model	Environment model	World model
(Conditional) exchangeability	Unconfoundedness	Ignorability, no unmeasured/residual confounding
Positivity	Feasibility	Experimental treatment assignment, common support, overlap
Treatment effect estimation using observational data	Off-policy evaluation	

Supplementary Table 2: List with collected items per study. ICU=intensive care unit, IPTW=inverse=probability-of-treatment weighting, TMLE=targeted minimum loss-based estimation, RL=reinforcement learning, NA=not applicable, NIV=non-invasive ventilation, MV=mechanical ventilation, VFD=ventilator-free day, AKI=acute kidney injury, RRT=renal replacement therapy.

Reference	**Studied treatment**	**Primary outcome**	**Number of included ICUs**	**Usage of open source databases**	**Study size (n patients)**	**Studied Treatment regime type**	**Used method**
Agodi 2017	Protocol compliance	Hospital-acquired complications	17	None	1,840	Static	parametric G formula
Althoff 2020	NIV	Need for MV	682	None	53,654	Static	IPTW
Amer 2021	Anti-inflammatory drugs	VFDs	168	None	860	Static	IPTW
Arabi 2018	Anti-inflammatory drugs	Mortality	14	None	309	Static	IPTW
Arabi 2020	Antimicrobials	Mortality	14	None	349	Static	IPTW
Arnaud 2020	Antimicrobials	AKI	1	MIMIC-III	26,865	Static	IPTW
Bailly 2015	Antimicrobials	Mortality	87	None	647	Static	IPTW
Bekaert 2011	Hospital-acquired complications	Mortality	32	None	4,479	Static	IPTW
Bologheanu 2023	Anti-inflammatory drugs	Mortality	1	AmsterdamUMCdb	2,946	Dynamic	RL
Chen 2021	Anti-inflammatory drugs	Mortality	1	None	428	Static	IPTW
Cheng 2019	Ordering of labs	Combined	1	MIMIC-III	6,060	Dynamic	RL
De Bus 2020	Antimicrobials	Clinical cure	152	None	1,495	Static	IPTW
Delaney 2016	Anti-inflammatory drugs	Mortality	51	None	607	Static	IPTW
Dupuis 2017	Blood transfusion	Mortality	23	None	6,016	Static	IPTW
Eghbali 2021	Sedatives & analgesics	Maintenance of clinical target value	1	MIMIC-IV	1,757	Dynamic	RL
Esperatti 2013	Multiple	Mortality	6	None	335	Static	IPTW
Frencken 2018	Bacterial colonization	Hospital-acquired complications	1	None	2,066	Static	IPTW
Guo 2022	Multiple	Mortality	1	MIMIC-III	13,762	Dynamic	RL
Huang 2022	Sodium bicarbonate	Mortality	1	MIMIC-IV	869	Static	IPTW
Jeter 2021	Vasopressors & IV fluids	Combined	1	MIMIC-III	5,366	Dynamic	RL

Kaushik 2022	Vasopressors & IV fluids	Combined	1	MIMIC-III	17,898	Dynamic	RL
Khanal 2012	RRT	Mortality	1	None	146	Static	IPTW
Klouwenberg 2014	Hospital-acquired complications	Mortality	1	None	1,112	Static	IPTW
Komorowski 2018	Vasopressors & IV fluids	Mortality	209	MIMIC-III + eICU	96,156	Dynamic	RL
Kondrup 2022	MV	Combined	1	MIMIC-III	61,532	Dynamic	RL
Li 2019	gastric acid-suppressing agents	Hospital-acquired complications	1	None	6,133	Static	parametric G formula
Li 2020	Anti-inflammatory drugs	Mortality	10	None	294	Static	IPTW
Libório 2020	Diuretics	Mortality	1	MIMIC-III	14,896	Static	IPTW
Lin 2018	Anticoagulants	Maintenance of clinical target value	2	MIMIC-III	4,908	Dynamic	RL
Liu 2016	Multiple	Vital signs	1	None	300	Static	parametric G formula
Lopez-Martinez 2019	Sedatives & analgesics	Combined	1	MIMIC-III	6,843	Dynamic	RL
Martucci 2023	Blood transfusion	Mortality	41	None	604	Dynamic	IPTW
Mecklenburg 2021	therapeutic hypothermia (TH)	Major bleeding	1	None	66	Static	IPTW
Mollura 2022	Vasopressors & IV fluids	Mortality	1	MIMIC-III	20,496	Dynamic	RL
Moromizato 2023	Anti-inflammatory drugs	Mortality	438	None	67,348	Static	IPTW
Morzywołek 2022	RRT	Mortality	1	None	13,403	Dynamic	IPTW
Muriel 2015	Sedatives & analgesics	Need for MV	322	None	842	Static	IPTW
Nemati 2016	Anticoagulants	Maintenance of clinical target value	1	MIMIC-II	4,470	Dynamic	RL
Ohbe 2018	Nutrition	Mortality	1200	None	1,769	Static	IPTW
Ong 2015	Hospital-acquired complications	Mortality	2	None	3,080	Static	IPTW
Ong 2016	Hospital-acquired complications	Mortality	2	None	399	Static	IPTW
Padmanabhan 2015	Sedatives & analgesics	Deviation from vital signs target value	simulated data	None	simulated data	Dynamic	RL
Padmanabhan 2017	Sedatives & analgesics	Maintenance of clinical target value	simulated data	None	simulated data	Dynamic	RL
Peine 2021	MV	Mortality	209	MIMIC-III + eICU	37,029	Dynamic	RL

Peng 2018	Vasopressors & IV fluids	Mortality	1	MIMIC-III	15,415	Dynamic	RL
Peng 2023	tracheostomy	Mortality	209	MIMIC-IV + eICU	626	Static	IPTW
Petersen 2019	Anti-inflammatory drugs	Mortality	simulated data	None	simulated data	Dynamic	RL
Pisani 2015	antipsychotic	Delirium	1	None	93	Static	IPTW
Pouwels 2017	Hospital-acquired complications	Mortality	2	None	3,411	Static	IPTW
Pouwels 2018	Hospital-acquired complications	Mortality	2	None	2,914	Static	IPTW
Pouwels 2020	Hospital-acquired complications	ICU LOS	2	None	2,914	Static	IPTW
Prasad 2017	MV	Combined	1	MIMIC-III	8,182	Dynamic	RL
Prasad 2022	Electrolyte replacement therapy	Combined	4	MIMIC-IV	53,234	Dynamic	RL
Raghu 2017	Vasopressors & IV fluids	Combined	1	MIMIC-III	17,898	Dynamic	RL
Raghu 2018	Vasopressors & IV fluids	Combined	1	MIMIC-III	17,898	Dynamic	RL
Ribba 2022	Sedatives & analgesics	Maintenance of clinical target value	simulated data	None	simulated data	Dynamic	RL
Roggeveen 2021	Vasopressors & IV fluids	Mortality	2	MIMIC-III + AmsterdamUMCdb	11,382	Dynamic	RL
Shahn 2020	IV fluids	Mortality	1	MIMIC-III	1,639	Dynamic	IPTW
Shahn 2021	Diuretics	Mortality	1	MIMIC-III	1,501	Dynamic	IPTW
Shahn 2023	MV	Mortality	1	MIMIC-IV	7,433	Dynamic	IPTW
Sinzinger 2005	Sedatives & analgesics	Maintenance of clinical target value	simulated data	None	simulated data	Dynamic	RL
Steen 2021	Hospital-acquired complications	Mortality	1	None	2,720	Static	IPTW
Su 2022	IV fluids	Mortality	1	None	2,705	Dynamic	RL
Tacquard 2021	Anticoagulants	Thrombotic complications	8	None	538	Static	IPTW
Torres 2020	ARDS	Mortality	3	None	658	Static	TMLE
Truche 2016	RRT	Mortality	19	None	1,360	Static	IPTW
Urner 2022	ECMO	Mortality	310	None	7,345	Dynamic	IPTW
Wang 2011	MV	Mortality	10	None	1,410	Dynamic	parametric G formula
Wang, Y. 2022	Blood transfusion	Combined	2	MIMIC-III	17,608	Dynamic	RL
Wang, Z. 2022	Vasopressors & IV fluids	Combined	1	MIMIC-IV	6,660	Dynamic	RL
Weng 2017	Glucose levels	Mortality	1	MIMIC-III	5,565	Dynamic	RL
Yang 2022	Dry weight	Combined	1	None	750	Dynamic	RL

Yarnell 2023	MV	Mortality	2	MIMIC-IV + AmsterdamUMCdb	4,636	Dynamic	parametric G formula
Zhang, L. 2021	Anti-inflammatory drugs	Mortality	208	eICU	1,557	Static	IPTW
Zhang, Q. 2023	Vasopressors & IV fluids	Combined	1	MIMIC-III	19,620	Dynamic	RL
Zhang, R. 2021	Diuretics	Mortality	20	None	932	Static	IPTW
Zhang, Z. 2018	Sodium bicarbonate	Mortality	1	MIMIC-III	1,718	Static	IPTW
Zhang, Z. 2019	Sodium bicarbonate	Mortality	1	MIMIC-III	3,406	Static	IPTW
Zheng 2021	NIV	Mortality	1	None	1,372	Dynamic	RL

Supplementary Table 3: Subcomponent-specific results of the quality of reporting assessment in the reproducibility domain, specifically for the studies inverse-probability-of-treatment weighting or targeted minimum loss-based estimation (n=43).

**Reference**	**Eligibility criteria**	**Treatment strategies**	**Outcome**	**Follow-up period**			**Analysis plan**	
				**Time zero**	**End of follow-up**	**Time resolution**	**Propensity score estimator**	**Propensity score predictors**
**Althoff 2020**	☺	☺	☺	☺	☺	☹	☹	☺
**Amer 2021**	☺	☹	☺	☺	☺	☺	☺	☺
**Arabi 2018**	☺	☹	☺	☺	☺	☺	☹	☺
**Arabi 2020**	☺	☹	☺	☺	☺	☺	☹	☺
**Arnaud 2020**	☺	☹	☺	☺	☺	☺	☺	☺
**Bailly 2015**	☺	☺	☺	☺	☺	☺	☺	☺
**Bekaert 2011**	☺	☺	☺	☺	☺	☺	☺	☺
**Chen 2021**	☺	☹	☺	☺	☺	☺	☹	☺
**De Bus 2020**	☺	☺	☺	☺	☺	☺	☺	☺
**Delaney 2016**	☺	☹	☺	☺	☺	☺	☺	☺
**Dupuis 2017**	☺	☺	☺	☺	☹	☺	☺	☺
**Esperatti 2013**	☺	☹	☺	☺	☺	☹	☺	☺
**Frencken 2018**	☺	☺	☺	☺	☺	☺	☹	☺
**Huang 2022**	☺	☺	☺	☺	☺	☹	☹	☺
**Khanal 2012**	☺	☺	☺	☹	☺	☺	☺	☺
**Klouwenberg 2014**	☺	☺	☺	☺	☺	☺	☺	☺
**Li 2020**	☺	☹	☺	☺	☺	☺	☹	☺
**Libório 2020**	☺	☹	☺	☺	☺	☺	☺	☺
**Martucci 2023**	☺	☺	☺	☺	☺	☺	☹	☺
**Mecklenburg 2021**	☺	☺	☺	☺	☺	☺	☺	☺
**Moromizato 2023**	☺	☺	☺	☺	☺	☺	☹	☹

**Morzywołek 2022**	☺	☺	☺	☺	☺	☺	☺	☺
**Muriel 2015**	☺	☺	☺	☺	☺	☺	☺	☺
**Ohbe 2018**	☺	☺	☺	☺	☺	☺	☺	☺
**Ong 2015**	☺	☺	☺	☺	☺	☺	☺	☺
**Ong 2016**	☺	☺	☺	☺	☺	☺	☺	☺
**Peng 2023**	☺	☺	☺	☺	☹	☺	☹	☺
**Pisani 2015**	☺	☺	☺	☺	☺	☺	☺	☺
**Pouwels 2017**	☺	☺	☺	☺	☺	☺	☺	☺
**Pouwels 2018**	☺	☺	☺	☺	☺	☺	☺	☺
**Pouwels 2020**	☺	☺	☺	☺	☺	☺	☺	☺
**Shahn 2020**	☺	☺	☺	☺	☺	☺	☺	☺
**Shahn 2021**	☺	☺	☺	☺	☺	☺	☺	☺
**Shahn 2023**	☺	☺	☺	☺	☺	☺	☺	☺
**Steen 2021**	☺	☺	☺	☺	☺	☺	☺	☺
**Tacquard 2021**	☺	☺	☺	☺	☺	☺	☹	☺
**Torres 2020**	☺	☺	☺	☺	☺	☺	☺	☺
**Truche 2016**	☺	☺	☺	☺	☺	☺	☺	☺
**Urner 2022**	☺	☺	☺	☺	☺	☺	☹	☺
**Zhang, L. 2021**	☺	☹	☺	☺	☺	☹	☺	☺
**Zhang, R. 2021**	☺	☺	☺	☺	☺	☺	☹	☹
**Zhang, Z. 2018**	☺	☺	☺	☺	☺	☺	☹	☺
**Zhang, Z. 2019**	☺	☹	☺	☺	☺	☺	☺	☺

Supplementary Table 4: Subcomponent-specific results of the quality of reporting assessment in the reproducibility domain, specifically for the studies using the parametric G formula (n=5).

**Reference**	**Eligibility criteria**	**Treatment strategies**	**Outcome**	**Follow-up period**			**Analysis plan (parametric G formula)**				
				**Time zero**	**End of follow-up**	**Time resolution**	**Outcome estimator**	**Outcome predictors**	**Confounders estimators**	**Confounders predictors**	**Method to evaluate the G formula**
**Agodi 2017**	☺	☺	☺	☺	☺	☺	☺	☺	☺	☺	☺
**Li 2019**	☺	☺	☺	☺	☺	☺	☺	☺	☺	☺	☺
**Liu 2016**	☺	☺	☺	☺	☺	☺	☺	☺	☹	☹	☹
**Wang 2011**	☺	☺	☺	☺	☺	☺	☺	☺	☺	☺	☺
**Yarnell 2023**	☺	☺	☺	☺	☺	☺	☺	☺	☺	☺	☺

Supplementary Table 5: Subcomponent-specific results of the quality of reporting assessment in the reproducibility domain, specifically for the studies using reinforcement learning (n=31). NA=not applicable

Reference	Eligibility criteria	Treatment strategies	Outcome	Follow-up period			Analysis plan (RL)			
				Time zero	End of follow-up	Time resolution	Learning scheme	State space model	Environment model	Discount factor
Bologheanu 2023	☺	☺	☹	☺	☺	☺	☺	☺	☺	☹
Cheng 2019	☺	☺	☺	☺	☺	☺	☺	☺	☺	☺
Eghbali 2021	☺	☺	☺	☺	☺	☺	☺	☺	☺	☺
Guo 2022	☺	☺	☺	☺	☺	☺	☺	☺	☺	☺
Jeter 2021	☺	☺	☺	☺	☺	☺	☺	☺	☺	☹
Kaushik 2022	☺	☺	☺	☹	☺	☺	☺	☺	☺	☹
Komorowski 2018	☺	☺	☺	☺	☺	☺	☺	☺	☺	☺
Kondrup 2022	☹	☺	☺	☺	☺	☺	☺	☺	☺	☺
Lin 2018	☺	☺	☺	☹	☹	☺	☺	☹	☺	☹
Lopez-Martinez 2019	☺	☺	☺	☹	☹	☺	☺	☺	☺	☹
Mollura 2022	☺	☹	☺	☺	☺	☺	☺	☺	☺	☺
Nemati 2016	☺	☺	☺	☺	☺	☺	☺	☺	☺	☹
Padmanabhan 2015	☺	☺	☺	NA	NA	NA	☺	☺	☺	☹
Padmanabhan 2017	☺	☺	☺	NA	NA	NA	☺	☺	☺	☹
Peine 2021	☺	☺	☺	☺	☺	☺	☺	☺	☺	☺
Peng 2018	☺	☺	☺	☹	☹	☺	☺	☺	☺	☺
Petersen 2019	☺	☺	☺	NA	NA	NA	☺	☺	☺	☺
Prasad 2017	☺	☺	☺	☺	☺	☺	☺	☺	☺	☹
Prasad 2022	☺	☺	☺	☺	☺	☺	☺	☹	☺	☹
Raghu 2017	☺	☺	☺	☺	☺	☺	☺	☺	☺	☹
Raghu 2018	☺	☺	☺	☺	☺	☺	☺	☹	☺	☹
Ribba 2022	☺	☹	☺	NA	NA	NA	☺	☺	☺	☺
Roggeveen 2021	☺	☺	☺	☺	☺	☺	☺	☺	☺	☹
Sinzinger 2005	☺	☺	☺	NA	NA	NA	☺	☺	☺	☺
Su 2022	☺	☺	☺	☹	☺	☺	☺	☹	☺	☹
Wang, Y. 2022	☺	☺	☺	☺	☺	☺	☺	☺	☺	☺
Wang, Z. 2022	☺	☺	☺	☺	☺	☺	☺	☺	☺	☹
Weng 2017	☺	☹	☺	☹	☹	☺	☺	☹	☹	☺
Yang 2022	☺	☺	☺	☺	☺	☹	☺	☺	☺	☺
Zhang, Q. 2023	☺	☺	☺	☺	☺	☺	☺	☺	☺	☹
Zheng 2021	☺	☺	☺	☺	☺	☹	☺	☹	☺	☺

Supplementary Table 6: reporting of assumptions assessment results per study. IPT=inverse probability of treatment

Reference	Conditional exchangeability			Positivity		Consistency
	Mentioned	Check for potential violations reported		Mentioned	Check for potential violations reported	Mentioned
		Indirect method	Bias analysis		Examination of IPT weights distribution	
Agodi 2017	☺	☹	☹	☹	☹	☹
Althoff 2020	☺	☺	☺	☹	☹	☹
Amer 2021	☺	☹	☹	☹	☹	☹
Arabi 2018	☺	☺	☹	☹	☹	☹
Arabi 2020	☺	☺	☹	☹	☹	☹
Arnaud 2020	☺	☹	☹	☹	☺	☹
Bailly 2015	☺	☺	☹	☺	☺	☺
Bekaert 2011	☺	☹	☹	☹	☹	☹
Bologheanu 2023	☹	☹	☹	☹	☹	☹
Chen 2021	☺	☹	☹	☹	☺	☹
Cheng 2019	☹	☹	☹	☹	☹	☹
De Bus 2020	☺	☺	☹	☺	☺	☹
Delaney 2016	☺	☹	☹	☹	☺	☹
Dupuis 2017	☺	☹	☺	☺	☺	☹
Eghbali 2021	☺	☹	☹	☹	☹	☹
Esperatti 2013	☹	☹	☹	☹	☹	☹
Frencken 2018	☺	☹	☹	☹	☹	☹
Guo 2022	☺	☹	☹	☹	☹	☺
Huang 2022	☺	☹	☹	☹	☹	☹
Jeter 2021	☹	☹	☹	☹	☹	☹
Kaushik 2022	☹	☹	☹	☹	☹	☹
Khanal 2012	☺	☹	☹	☺	☺	☹
Klouwenberg 2014	☺	☺	☹	☹	☹	☹
Komorowski 2018	☹	☹	☹	☹	☹	☹
Kondrup 2022	☹	☹	☹	☹	☹	☹
Li 2019	☺	☹	☹	☹	☹	☹
Li 2020	☹	☹	☹	☹	☺	☹
Libório 2020	☺	☹	☹	☺	☺	☹
Lin 2018	☺	☹	☹	☹	☹	☹
Liu 2016	☺	☹	☹	☹	☹	☺
Lopez-Martinez 2019	☹	☹	☹	☹	☹	☹
Martucci 2023	☺	☹	☹	☹	☹	☹
Mecklenburg 2021	☺	☹	☹	☹	☹	☹
Mollura 2022	☹	☺	☹	☹	☹	☹
Moromizato 2023	☺	☹	☺	☹	☹	☹
Morzywołek 2022	☺	☹	☹	☹	☺	☹
Muriel 2015	☺	☹	☹	☹	☹	☹
Nemati 2016	☹	☹	☹	☹	☹	☹
Ohbe 2018	☺	☹	☺	☹	☹	☹
Ong 2015	☺	☹	☹	☹	☹	☹
Ong 2016	☺	☺	☹	☹	☹	☹
Padmanabhan 2015	☹	☹	☹	☹	☹	☹
Padmanabhan 2017	☹	☹	☹	☹	☹	☹
Peine 2021	☺	☹	☹	☹	☹	☹
Peng 2018	☺	☹	☹	☹	☺	☹
Peng 2023	☺	☺	☺	☹	☹	☹
Petersen 2019	☹	☹	☹	☹	☹	☹
Pisani 2015	☺	☹	☹	☹	☹	☹
Pouwels 2017	☺	☹	☹	☹	☺	☹
Pouwels 2018	☺	☺	☹	☹	☺	☹
Pouwels 2020	☺	☺	☹	☺	☺	☺

**Prasad 2017**	☹	☹	☹	☹	☹	☹
**Prasad 2022**	☺	☹	☹	☹	☹	☹
**Raghu 2017**	☺	☹	☹	☺	☹	☹
**Raghu 2018**	☺	☹	☹	☹	☹	☹
**Ribba 2022**	☹	☹	☹	☹	☹	☹
**Roggeveen 2021**	☹	☹	☹	☹	☹	☹
**Shahn 2020**	☺	☺	☹	☺	☹	☺
**Shahn 2021**	☺	☺	☹	☺	☺	☹
**Shahn 2023**	☺	☹	☹	☺	☹	☹
**Sinzinger 2005**	☹	☹	☹	☹	☹	☹
**Steen 2021**	☺	☹	☹	☺	☺	☺
**Su 2022**	☺	☹	☹	☹	☹	☹
**Tacquard 2021**	☹	☹	☹	☹	☹	☹
**Torres 2020**	☹	☺	☹	☹	☹	☹
**Truche 2016**	☺	☹	☺	☺	☺	☺
**Urner 2022**	☺	☹	☺	☹	☹	☹
**Wang 2011**	☺	☹	☹	☺	☹	☺
**Wang, Y. 2022**	☹	☹	☹	☹	☹	☹
**Wang Z. 2022**	☹	☹	☹	☹	☹	☹
**Weng 2017**	☹	☹	☹	☹	☹	☹
**Yang 2022**	☺	☹	☹	☹	☹	☹
**Yarnell 2023**	☺	☹	☺	☺	☹	☺
**Zhang, L. 2021**	☹	☹	☹	☹	☹	☹
**Zhang, Q. 2023**	☹	☹	☹	☹	☹	☹
**Zhang, R. 2021**	☹	☹	☹	☹	☺	☹
**Zhang, Z. 2018**	☺	☹	☹	☹	☺	☹
**Zhang, Z. 2019**	☺	☹	☹	☹	☺	☹
**Zheng 2021**	☹	☹	☹	☹	☹	☹

Supplementary Table 7: filled PRISMA Extension for Scoping Reviews (PRISMAScR) checklist.

SECTION	ITEM	PRISMA-ScR CHECKLIST ITEM	REPORTED ON PAGE #
**TITLE**			
**Title**	1	Identify the report as a scoping review.	1
**ABSTRACT**			
**Structured summary**	2	Provide a structured summary that includes (as applicable): background, objectives, eligibility criteria, sources of evidence, charting methods, results, and conclusions that relate to the review questions and objectives.	3
**INTRODUCTION**			
**Rationale**	3	Describe the rationale for the review in the context of what is already known. Explain why the review questions/objectives lend themselves to a scoping review approach.	8-10
**Objectives**	4	Provide an explicit statement of the questions and objectives being addressed with reference to their key elements (e.g., population or participants, concepts, and context) or other relevant key elements used to conceptualize the review questions and/or objectives.	10
**METHODS**			
**Protocol and registration**	5	Indicate whether a review protocol exists; state if and where it can be accessed (e.g., a Web address); and if available, provide registration information, including the registration number.	22
**Eligibility criteria**	6	Specify characteristics of the sources of evidence used as eligibility criteria (e.g., years considered, language, and publication status), and provide a rationale.	22-23
**Information sources***	7	Describe all information sources in the search (e.g., databases with dates of coverage and contact with authors to identify additional sources), as well as the date the most recent search was executed.	22
**Search**	8	Present the full electronic search strategy for at least 1 database, including any limits used, such that it could be repeated.	Supplementary Table 8
**Selection of sources of evidence†**	9	State the process for selecting sources of evidence (i.e., screening and eligibility) included in the scoping review.	23
**Data charting process‡**	10	Describe the methods of charting data from the included sources of evidence (e.g., calibrated forms or forms that have been tested by the team before their use, and whether data charting was done independently or in duplicate) and any processes for obtaining and confirming data from investigators.	23
**Data items**	11	List and define all variables for which data were sought and any assumptions and simplifications made.	24
**Critical appraisal of individual**	12	If done, provide a rationale for conducting a critical appraisal of included sources of evidence;	24-25

sources of evidence§		describe the methods used and how this information was used in any data synthesis (if appropriate).	
Synthesis of results	13	Describe the methods of handling and summarizing the data that were charted.	26
**RESULTS**			
Selection of sources of evidence	14	Give numbers of sources of evidence screened, assessed for eligibility, and included in the review, with reasons for exclusions at each stage, ideally using a flow diagram.	Figure 2
Characteristics of sources of evidence	15	For each source of evidence, present characteristics for which data were charted and provide the citations.	Table 2 and Supplementary Table 2
Critical appraisal within sources of evidence	16	If done, present data on critical appraisal of included sources of evidence (see item 12).	11-13
Results of individual sources of evidence	17	For each included source of evidence, present the relevant data that were charted that relate to the review questions and objectives.	Supplementary Figures 3-8
Synthesis of results	18	Summarize and/or present the charting results as they relate to the review questions and objectives.	Figure 3
**DISCUSSION**			
Summary of evidence	19	Summarize the main results (including an overview of concepts, themes, and types of evidence available), link to the review questions and objectives, and consider the relevance to key groups.	13-15
Limitations	20	Discuss the limitations of the scoping review process.	21
Conclusions	21	Provide a general interpretation of the results with respect to the review questions and objectives, as well as potential implications and/or next steps.	22
**FUNDING**			
Funding	22	Describe sources of funding for the included sources of evidence, as well as sources of funding for the scoping review. Describe the role of the funders of the scoping review.	26

JBI = Joanna Briggs Institute; PRISMA-ScR = Preferred Reporting Items for Systematic reviews and Meta-Analyses extension for Scoping Reviews.
* Where *sources of evidence* (see second footnote) are compiled from, such as bibliographic databases, social media platforms, and Web sites.
† A more inclusive/heterogeneous term used to account for the different types of evidence or data sources (e.g., quantitative and/or qualitative research, expert opinion, and policy documents) that may be eligible in a scoping review as opposed to only studies. This is not to be confused with *information sources* (see first footnote).
‡ The frameworks by Arksey and O'Malley (6) and Levac and colleagues (7) and the JBI guidance (4, 5) refer to the process of data extraction in a scoping review as data charting.
§ The process of systematically examining research evidence to assess its validity, results, and relevance before using it to inform a decision. This term is used for items 12 and 19 instead of "risk of bias" (which is more applicable to systematic reviews of interventions) to include and acknowledge the various sources of evidence that may be used in a scoping review (e.g., quantitative and/or qualitative research, expert opinion, and policy document).

From: Tricco AC, Lillie E, Zarin W, O'Brien KK, Colquhoun H, Levac D, et al. PRISMA Extension for Scoping Reviews (PRISMAScR): Checklist and Explanation. Ann Intern Med. 2018;169:467–473. doi: 10.7326/M18-0850.

Supplementary Table 8: Literature search strategies per database.

Database	Search Strategy
**Embase.com**	('causal inference'/de OR 'causal model'/de OR 'causal modeling'/de OR 'inverse probability weighting'/de OR ((causal NEAR/3 (inferen* OR model*)) OR ((causal OR average-treatment* OR individuali*-treatment* OR personali*-treatment*) NEXT/1 (effect*)) OR time-vary*-confound* OR g-computation* OR g-estimation* OR g-formula* OR doubly-robust OR counterfactual* OR (inverse-probabilit* NEAR/3 (weight* OR estimat*)) OR ((marginal-structur* OR structural-nest* OR causal-effect* OR causal-graphic* OR causal-inferen* OR condition*-outcome* OR sequen*-cox*) NEAR/3 (method* OR model*)) OR TAR-Net OR (Treatment*-Agnost* NEAR/3 Representat* NEAR/3 Network*) OR double-machine-learning OR causal-forest* OR deconfoun* OR anchor*-regress* OR x-learner* OR t-learner* OR s-learner* OR q-learning OR q-network OR reinforcement*-learn* OR ((policy OR value) NEXT/1 iteration*) OR temporal-differen* OR actor-critic* OR (Markov NEAR/3 decision NEAR/3 process*)):ab,ti) AND ('intensive care'/exp OR 'intensive care unit'/exp OR 'critically ill patient'/de OR 'critical illness'/de OR 'artificial ventilation'/exp OR 'mechanical ventilator'/exp OR (intensive-care* OR critical-care* OR critical*-ill* OR icu OR ((mechanic* OR artificial*) NEAR/3 ventilat*)):Ab,ti,jt) NOT [conference abstract]/lim AND [english]/lim NOT ('pediatric intensive care unit'/de OR 'neonatal intensive care unit'/de OR child/exp OR pediatrics/exp OR (nicu OR picu OR nicus OR picus OR infant* OR child* OR neonat* OR newborn* OR pediatr* OR paediatr*):ab,ti)
**Medline ALL**	(((caus* ADJ3 (inferen* OR model*)) OR ((causal OR average-treatment* OR individuali*-treatment* OR personali*-treatment*) ADJ (effect* OR method*)) OR time-vary*-confound* OR g-computation* OR g-estimation* OR g-formula* OR doubly-robust-estimation* OR counterfactual* OR (inverse-probabilit* ADJ3 (weight* OR estimat*)) OR ((marginal-structur* OR structural-nest* OR causal-effect* OR causal-graphic* OR causal-inferen* OR semi-paramet* OR semiparamet* OR fully-paramet*) ADJ3 (method* OR model*)) OR TAR-Net OR (Treatment*-Agnost* ADJ3 Representat* ADJ3 Network*) OR double-machine-learning OR causal-forest* OR deconfoun* OR anchor*-regress* OR x-learner* OR t-learner* OR s-learner* OR q-learning OR q-network OR reinforcement*-learn* OR ((policy OR value) ADJ iteration*) OR temporal-differen* OR actor-critic* OR (Markov ADJ3 decision ADJ3 process*)).ab,ti. OR (RL OR IRL).ti.) AND (exp Intensive Care Units/ OR Critical Illness/ OR exp Respiration, Artificial/ OR exp Ventilators, Mechanical/ OR (intensive-care* OR critical-care*OR critical*-ill* OR icu OR ((mechanic* OR artificial*) ADJ3 ventilat*)).ab,ti,jt) NOT (conference abstract) AND english.la. NOT (Intensive Care Units, Pediatric/de OR Intensive Care Units, Neonatal/de OR exp Child/ OR exp pediatrics/ OR (nicu OR picu OR nicus OR picus OR infant* OR child* OR neonat* OR newborn* OR pediatr* OR paediatr*).ti,ab)
**Web of Science Core Collection**	TS=(((causal NEAR/2 (inferen* OR model*)) OR ((causal OR average-treatment* OR individuali*-treatment* OR personali*-treatment*) NEAR/1 (effect*)) OR time-vary*-confound* OR g-computation* OR g-estimation* OR g-formula* OR doubly-robust OR counterfactual* OR (inverse-probabilit* NEAR/2 (weight* OR estimat*)) OR ((marginal-structur* OR structural-nest* OR causal-effect* OR causal-graphic* OR causal-inferen* OR condition*-outcome* OR sequen*-cox*) NEAR/2 (method*

	OR model*)) OR TAR-Net OR (Treatment*-Agnost* NEAR/2 Representat* NEAR/2 Network*) OR double-machine-learning OR causal-forest* OR deconfoun* OR anchor*-regress* OR x-learner* OR t-learner* OR s-learner* OR q-learning OR q-network OR reinforcement*-learn* OR ((policy OR value) NEAR/1 iteration*) OR temporal-differen* OR actor-critic* OR (Markov NEAR/2 decision NEAR/2 process*)) AND (intensive-care* OR critical-care* OR critical*-ill* OR icu OR ((mechanic* OR artificial*) NEAR/2 ventilat*)) NOT (nicu OR picu OR nicus OR picus OR infant* OR child* OR neonat* OR newborn* OR pediatr* OR paediatr*)) AND DT=(Article OR Review OR Letter OR Early Access)
**Google Scholar**	Searched with 2 different queries:<br>• "causal inference"|"marginal structural models"|"g-formula"|"structural nested models"|"reinforcement learning" "intensive|critical care"<br><br>Only the <u>first 200</u> results<br><br>• "causal inference"|"marginal structural models"|"g-formula"|"structural nested models"|"reinforcement learning" intitle:"intensive|critical care"
**MedRxiv and BioRxiv**	Searched via Google with the following query:<br>inurl:medrxiv|biorxiv filetype:pdf "causal inference"|"marginal structural models"|"g-formula"|"structural nested models"|"reinforcement learning" "intensive|critical care"
**arXiv**	• Searched via Google with the following query:<br>inurl:arxiv filetype:pdf "causal inference"|"marginal structural models"|"g-formula"|"structural nested models"|"reinforcement learning" "intensive|critical care"<br><br>• Additionally, we searched through arXiv using 'advanced search'. We performed ten queries, searching with all possible combinations of the terms ["causal", "reinforcement learning"] and ["intensive care", "critical care", "icu", "mechanical ventilation", "critically ill"], combining these with an 'AND' statement, and selecting 'Abstract' (ie, this will identify articles that have this term in their abstract) for both terms.
**ACM Digital Library**	We searched through ACM Digital Library using 'advanced search'. Specifically, we combined two 'Search Within Abstract' terms, using the following terms:<br><br>• Term 1:<br>"causal inference" "causal effect" "causal model*" "inverse probability" "individualized treatment" "average treatment" "time \-varying confound*" "g computation" "g \-computation" "g estimation" "g \-estimation" "g formula" "g \-formula" "doubly robust" "doubly \-robust" "marginal structural" "structural nested" "TARNET" "double machine

	learning” “anchor regression” “x \-learner” “t \-learner” “s \-learner” “q \-learning” “q learning” “Q learning” “Q \-learning” “reinforcement learning” “temporal difference” “actor \-critic” “actor critic” “Markov decsion process” "causal forest" "deconfounder"<br><br>• Term 2:<br>“intensive care” “critical care” “critically ill” “critical ill*” “icu” “mechanical vent*” “artificial vent*”

Supplementary Table 9: Leading questions for each target trial framework subcomponent considered in the quality of reporting assessment. The analysis plan component is subdivided in specific subcomponents for each used causal inference method. IPTW=inverse probability of treatment weighting, TMLE= targeted minimum loss-based estimation, RL=reinforcement learning.

Component	**Subcomponent**	**Leading question**
Eligibility criteria	-	Are eligibility criteria for target population described?
Treatment strategies	-	Are the compared regimes described in such a way that one can think of an analogue randomized trial (ie, target trial)?
Outcome	-	Is the considered patient outcome described?
Follow-up period	time-zero	Is the time-zero (baseline) explicitly mentioned or can it reasonably be assumed from the data collection description?
	Follow-up	Are the start and end of follow-up period explicitly mentioned or can these reasonably be assumed from the data collection description?
	Time-resolution	Is the size of the considered time steps (ie, the time-resolution) explicitly mentioned or can it reasonably be assumed from the data collection description?
Analysis plan (RL)	Learning scheme	Is the learning scheme used to train the RL agent described?
	State space model	Does the methods description specify whether continuous or categorical state space and on which variables states were based?
	Environment model	Is the modelling of environment described (or clearly not applicable, eg, with model-free learning schemes)?
	Discount factor	Is the used discount factor described?
Analysis plan (parametric G formula)	Outcome estimator	Is the model used to estimate the outcome described? (eg, logistic regression)
	Outcome predictors	Are variables/features used to model the outcome described (including both time-fixed and time-varying variables)?
	Confounders estimators	Is the model used to estimate the confounders described? (eg, logistic regression)
	Confounders predictors	Are variables/features used to model the confounders described?
	Method to evaluate the G formula	Is the method to evaluate the G formula described? (eg, Monte-Carlo sampling)

Analysis plan (IPTW/TMLE)	Propensity score estimator	Is the model used to estimate the propensity score described? (eg, logistic regression)
	Propensity score predictors	Are variables/features used to model the propensity score described (including both time-fixed and time-varying variables)?

Supplementary Figures

Supplementary Figure 1: Figure S1: Bar chart representing the number of published articles using the different (a) modelling strategies and (b) studied treatment regimes over the years. IPTW=inverse probability-of-treatment weighting, RL=reinforcement learning, TMLE=targeted minimum loss- based estimation.

a

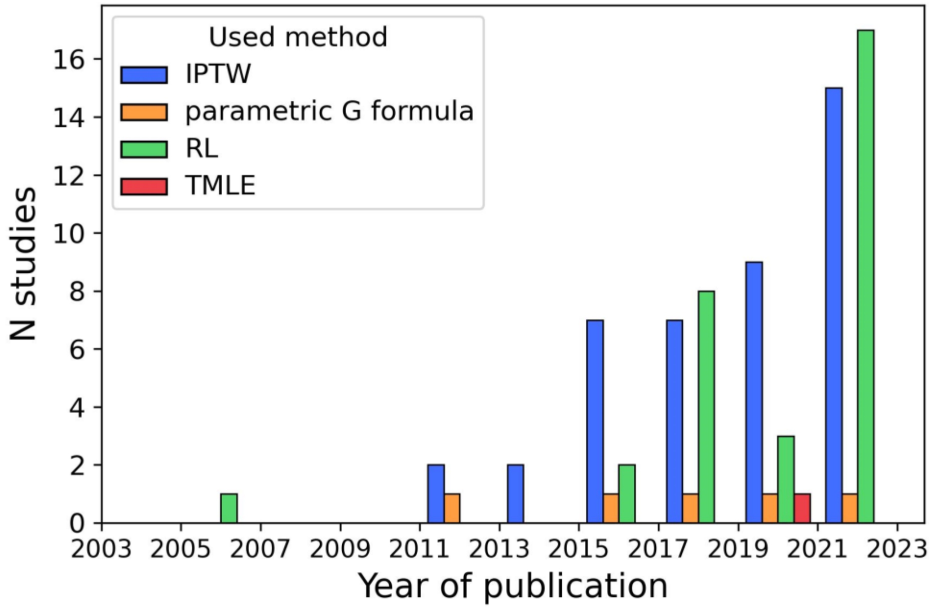

b

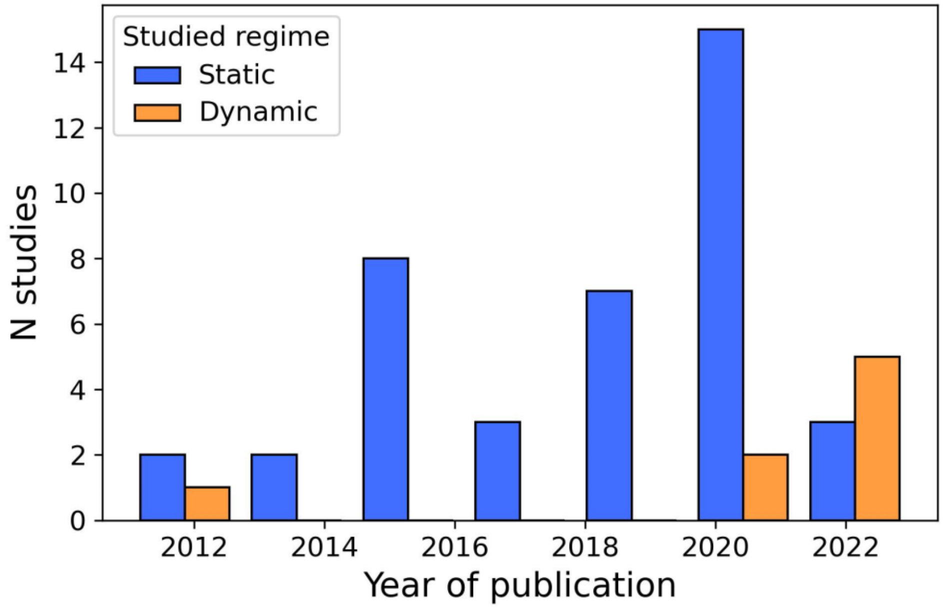

Supplementary Figure 2: Nested pie chart representing the off-policy evaluation (OPE) methods used in the reinforcement learning studies that used real patient data (n=26). IS=Importance sampling, MB=Model-based, DR=Doubly robust.

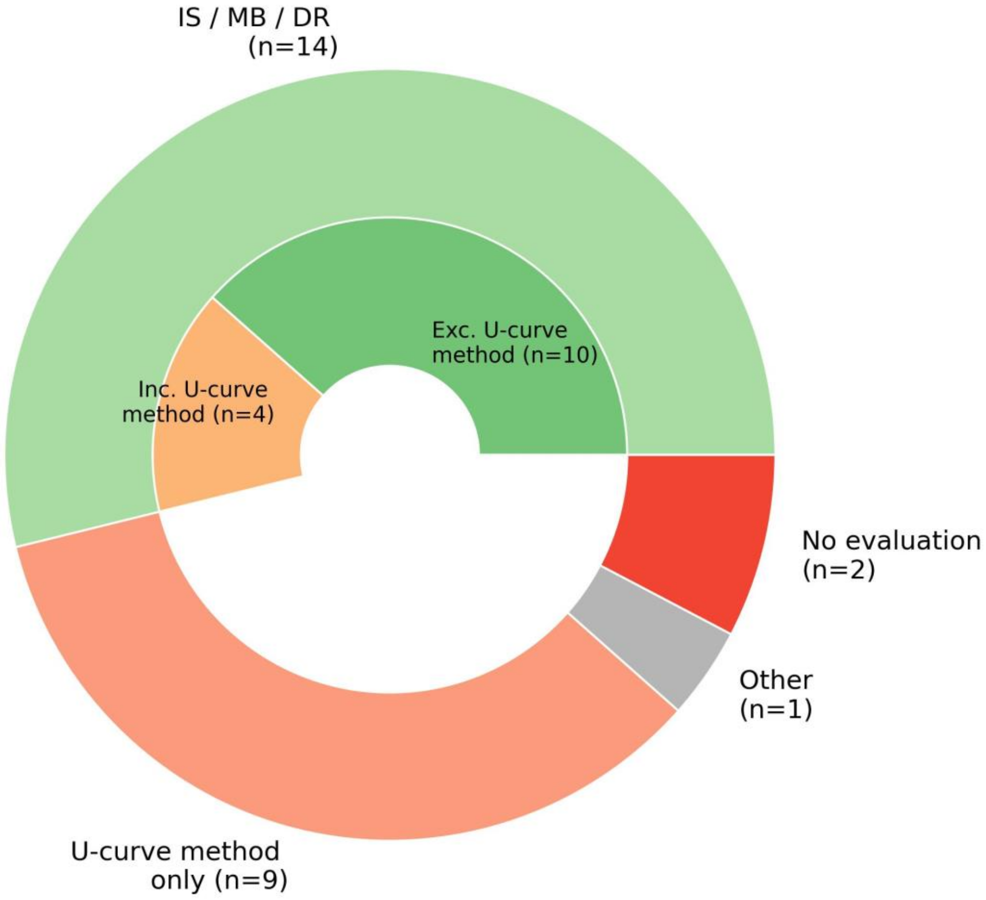

Supplementary Figure 3: Reporting of the target trial components in studies using inverse-probability-of-treatment weighting or targeted minimum loss-based estimation (n=43).

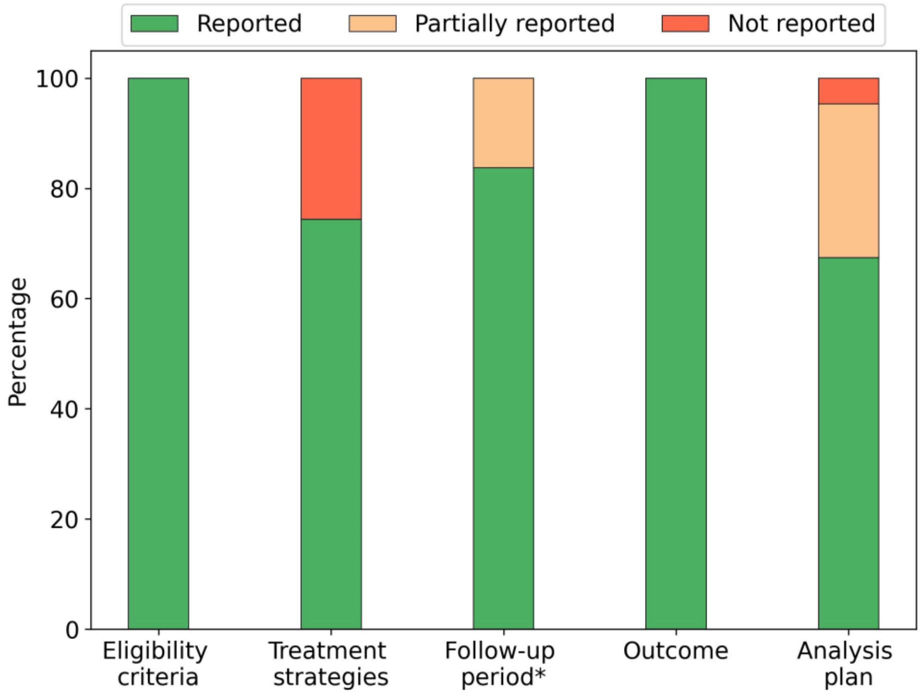

Supplementary Figure 4: Reporting of the target trial components in studies using the parametric G formula (n=5).

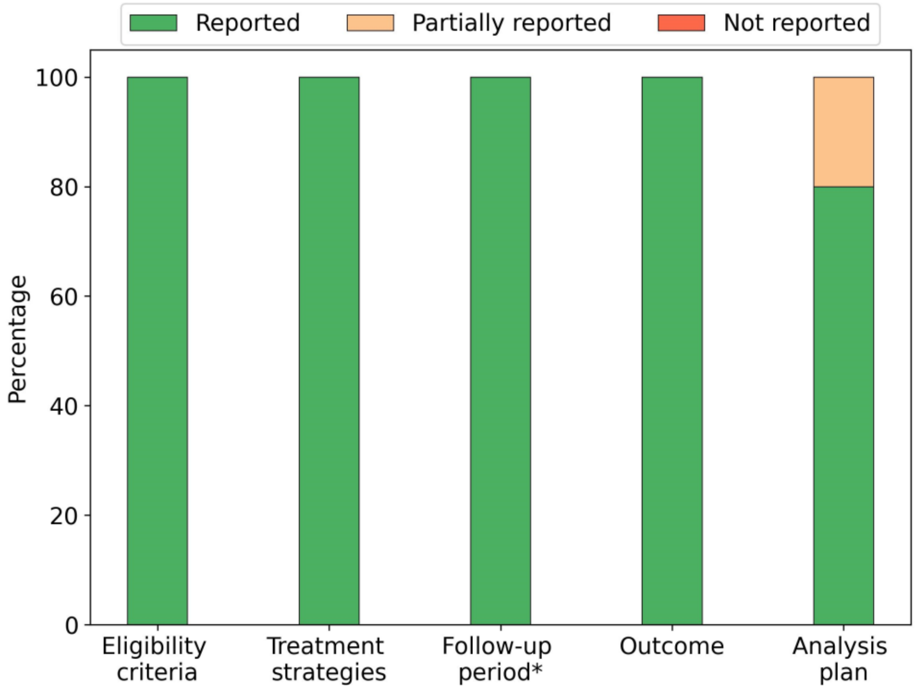

Supplementary Figure 5: Reporting of the target trial components in studies using reinforcement learning (n=28). *For the follow-up component, the studies that used simulated patient data (n=5) are not taken into account.

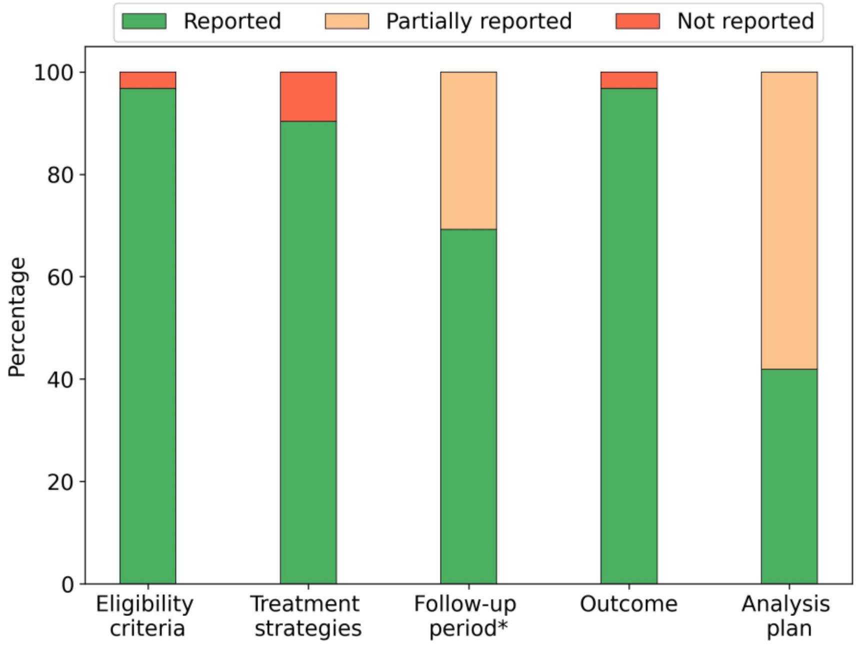

Supplementary Figure 6: Reporting of assumptions in the studies using inverse-probability-of-treatment weighting or targeted minimum loss-based estimation (n=43). Level 1=assumption not mentioned, level 2=assumption mentioned, level 3=attempt to check for potential violations of the assumption reported.

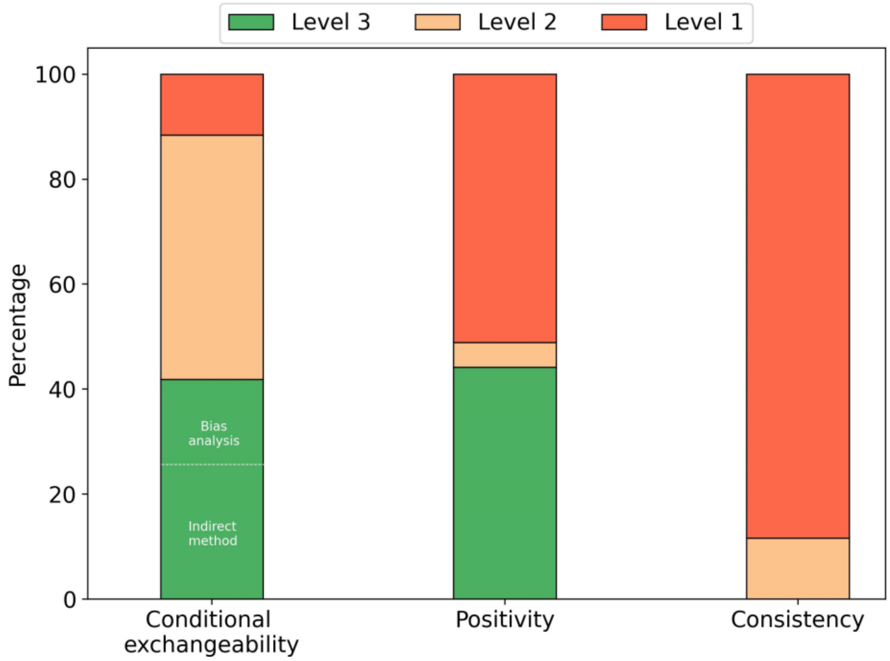

Supplementary Figure 7: Reporting of assumptions in the studies using the parametric G formula (n=5). Level 1=assumption not mentioned, level 2=assumption mentioned, level 3=attempt to check for potential violations of the assumption reported.

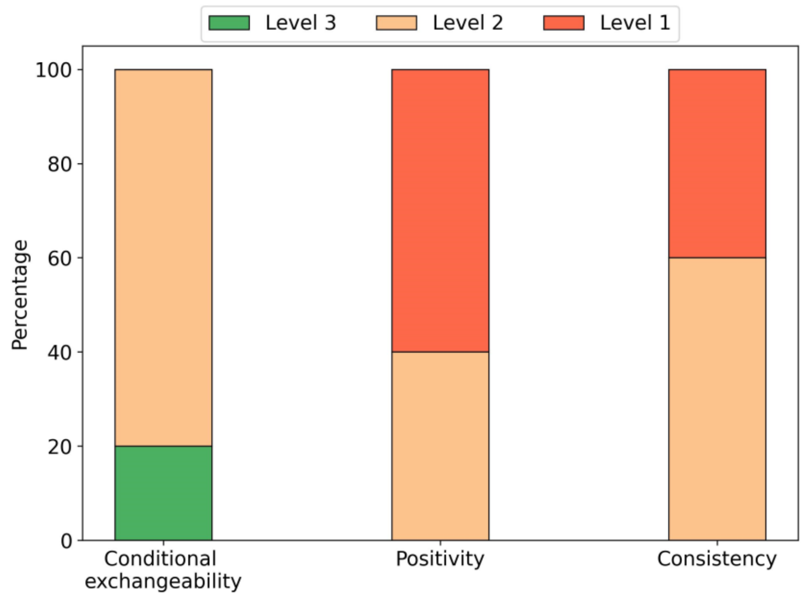

Supplementary Figure 8: Reporting of assumptions in the studies using reinforcement learning (n=31). Level 1=assumption not mentioned, level 2=assumption mentioned, level 3=attempt to check for potential violations of the assumption reported.

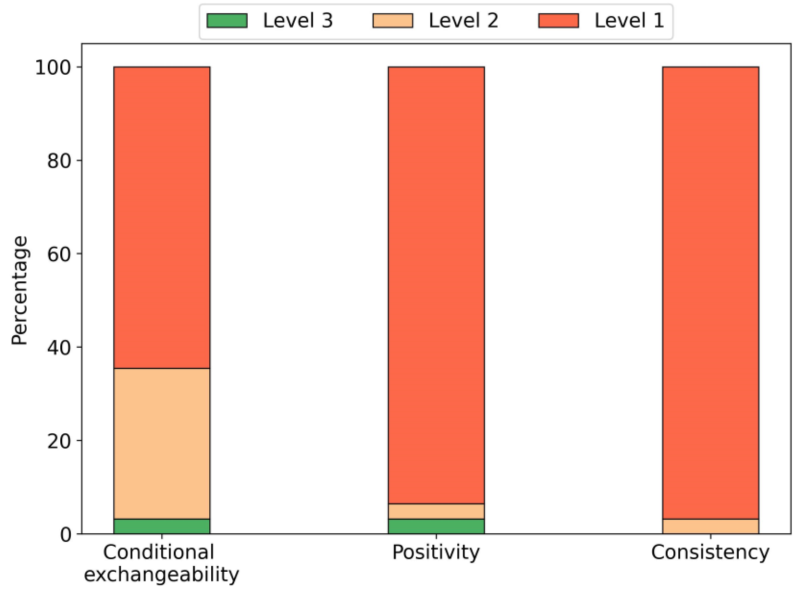

Supplementary References

1. Agodi, A. *et al.* Preventable proportion of intubation-associated pneumonia: Role of adherence to a care bundle. *PLoS One* **12**, (2017).
2. Althoff, M. D. *et al.* Noninvasive Ventilation Use in Critically Ill Patients with Acute Asthma Exacerbations. *Am. J. Respir. Crit. Care Med.* **202**, 1520–1530 (2020).
3. Amer, M. *et al.* Clinical characteristics and outcomes of critically ill mechanically ventilated COVID-19 patients receiving interleukin-6 receptor antagonists and corticosteroid therapy: a preliminary report from a multinational registry. *Eur. J. Med. Res.* **26**, 1–12 (2021).
4. Arabi, Y. M. *et al.* Corticosteroid therapy for critically ill patients with middle east respiratory syndrome. *Am. J. Respir. Crit. Care Med.* **197**, 757–767 (2018).
5. Arabi, Y. M. *et al.* Ribavirin and Interferon Therapy for Critically Ill Patients with Middle East Respiratory Syndrome: A Multicenter Observational Study. *Clin. Infect. Dis.* **70**, 1837–1844 (2020).
6. Arnaud, F. C. D. S. & Libório, A. B. Attributable nephrotoxicity of vancomycin in critically ill patients: A marginal structural model study. *J. Antimicrob. Chemother.* **75**, 1031–1037 (2020).
7. Bailly, S. *et al.* Failure of empirical systemic antifungal therapy in mechanically ventilated critically ill patients. *Am. J. Respir. Crit. Care Med.* **191**, 1139–1146 (2015).
8. Bekaert, M. *et al.* Attributable mortality of ventilator-associated pneumonia: A reappraisal using causal analysis. *Am J Respir Crit Care Med* **184**, 1133–1139 (2011).
9. Bologheanu, R. *et al.* Development of a Reinforcement Learning Algorithm to Optimize Corticosteroid Therapy in Critically Ill Patients with Sepsis. *J. Clin. Med.* **12**, (2023).
10. Chen, H. *et al.* Corticosteroid Therapy Is Associated With Improved Outcome in Critically Ill Patients With COVID-19 With Hyperinflammatory Phenotype. *Chest* **159**, 1793–1802 (2021).
11. Cheng, L.-F., Prasad, N. & Engelhardt, B. E. An Optimal Policy for Patient Laboratory Tests in Intensive Care Units. *Pac. Symp. Biocomput.* **24**, 320–331 (2019).
12. De Bus, L. *et al.* Antimicrobial de-escalation in the critically ill patient and assessment of clinical cure: the DIANA study. *Intensive Care Med.* **46**, 1404–1417 (2020).
13. Delaney, J. W. *et al.* The influence of corticosteroid treatment on the outcome of influenza A(H1N1pdm09)-related critical illness. *Crit. Care* **20**, (2016).
14. Dupuis, C. *et al.* Effect of transfusion on mortality and other adverse events among critically ill septic patients: An observational study using a marginal structural Cox model. *Crit. Care Med.* **45**, 1972–1980 (2017).
15. Eghbali, N., Alhanai, T. & Ghassemi, M. M. Patient-Specific Sedation Management via Deep Reinforcement Learning. *Front. Digit. Heal.* **3**, 608893 (2021).
16. Esperatti, M. *et al.* Validation of Predictors of Adverse Outcomes in Hospital-Acquired Pneumonia in the ICU. *Crit. Care Med.* **41**, 2151–2161 (2013).
17. Frencken, J. F. *et al.* Associations between enteral colonization with gram-negative bacteria and intensive care unit-acquired infections and colonization of the respiratory tract. *Clin. Infect. Dis.* **66**, 497–503 (2018).
18. Guo, H., Li, J., Liu, H. & He, J. Learning dynamic treatment strategies for coronary heart diseases

by artificial intelligence: real-world data-driven study. *BMC Med. Inform. Decis. Mak.* **22**, 1–16 (2022).
19. Huang, S. *et al.* Effectiveness of sodium bicarbonate infusion on mortality for elderly septic patients with acute metabolic acidosis. *Front. Pharmacol.* **13**, 1–8 (2022).
20. Jeter, R., Lehman, L.-W., Josef, C., Shashikumar, S. & Nemati, S. *Learning to Treat Hypotensive Episodes in Sepsis Patients Using a Counterfactual Reasoning Framework*. *Preprint at* https://www.medrxiv.org/content/early/2021/03/07/2021.03.03.21252863 (2021) doi:10.1101/2021.03.03.21252863.
21. Kaushik, P., Kummetha, S., Moodley, P. & Bapi, R. S. *A conservative q-learning approach for handling distribution shift in sepsis treatment strategies*. *Preprint at* https://arxiv.org/abs/2203.13884 (2022).
22. Khanal, N. *et al.* Comparison of outcomes by modality for critically ill patients requiring renal replacement therapy: A single-centre cohort study adjusting for time-varying illness severity and modality exposure. *Anaesth. Intensive Care* **40**, 260–268 (2012).
23. Klein Klouwenberg, P. M. *et al.* The attributable mortality of delirium in critically ill patients: prospective cohort study. *BMJ* **349**, g6652 (2014).
24. Komorowski, M., Celi, L. A., Badawi, O., Gordon, A. C. & Faisal, A. A. The Artificial Intelligence Clinician learns optimal treatment strategies for sepsis in intensive care. *Nat Med* **24**, 1716–1720 (2018).
25. Kondrup, F. *et al.* Towards Safe Mechanical Ventilation Treatment Using Deep Offline Reinforcement Learning. *Proc. AAAI Conf. Artif. Intell.* **37**, 15696–15702 (2023).
26. Li, X., Klompas, M., Menchaca, J. T. & Young, J. G. Effects of daily treatment with acid suppressants for stress ulcer prophylaxis on risk of ventilator-Associated events. *Infect Control Hosp Epidemiol* **41**, 187–193 (2019).
27. Li, Y. *et al.* Corticosteroid therapy in critically ill patients with COVID-19: a multicenter, retrospective study. *Crit. Care* **24**, (2020).
28. Libório, A. B., Barbosa, M. L., Sá, V. B. & Leite, T. T. Impact of loop diuretics on critically ill patients with a positive fluid balance. *Anaesthesia* **75**, e134–e142 (2020).
29. Lin, R., Stanley, M. D., Ghassemi, M. M. & Nemati, S. A Deep Deterministic Policy Gradient Approach to Medication Dosing and Surveillance in the ICU. *Conf Proc IEEE Eng Med Biol Soc.* (2018) doi:10.1109/EMBC.2018.8513203.A.
30. Liu, Q., Henry, K., Xu, Y. & Saria, S. Using Causal Inference to Estimate What-if Outcomes for Targeting Treatments. in *30th Conference on Neural Information Processing Systems* (2016).
31. Lopez-Martinez, D. *et al.* Deep Reinforcement Learning for Optimal Critical Care Pain Management with Morphine using Dueling Double-Deep Q Networks. *Conf. Proc. ... Annu. Int. Conf. IEEE Eng. Med. Biol. Soc. IEEE Eng. Med. Biol. Soc. Annu. Conf.* **2019**, 3960–3963 (2019).
32. Martucci, G. *et al.* Transfusion practice in patients receiving VV ECMO (PROTECMO): a prospective, multicentre, observational study. *Lancet. Respir. Med.* **11**, 245–255 (2023).
33. Mecklenburg, A. *et al.* Impact of therapeutic hypothermia on bleeding events in adult patients treated with extracorporeal life support peri-cardiac arrest. *J. Crit. Care* **62**, 12–18 (2021).
34. Mollura, M., Drudi, C., Lehman, L.-W. & Barbieri, R. A Reinforcement Learning Application for Optimal Fluid and Vasopressor Interventions in Septic ICU Patients. in *2022 44th Annual*

International Conference of the IEEE Engineering in Medicine & Biology Society (EMBC) 321–324 (2022). doi:10.1109/EMBC48229.2022.9871055.

35. Moromizato, T., Sakaniwa, R., Tokuda, Y., Taniguchi, K. & Shibuya, K. Intravenous methylprednisolone pulse therapy and the risk of in-hospital mortality among acute COVID-19 patients: Nationwide clinical cohort study. *Crit. Care* **27**, 1–11 (2023).
36. Morzywołek, P. *et al.* Timing of dialysis in acute kidney injury using routinely collected data and dynamic treatment regimes. *Crit. Care* **26**, 365 (2022).
37. Muriel, A. *et al.* Impact of sedation and analgesia during noninvasive positive pressure ventilation on outcome: a marginal structural model causal analysis. *Intensive Care Med* **41**, 1586–1600 (2015).
38. Nemati, S., Ghassemi, M. M. & Clifford, G. D. Optimal medication dosing from suboptimal clinical examples: a deep reinforcement learning approach. *Annu. Int. Conf. IEEE Eng. Med. Biol. Soc.* 2978–2981 (2016) doi:10.1109/EMBC.2016.7591355.
39. Ohbe, H. *et al.* Early enteral nutrition for cardiogenic or obstructive shock requiring venoarterial extracorporeal membrane oxygenation: a nationwide inpatient database study. *Intensive Care Med.* **44**, 1258–1265 (2018).
40. Ong, D. S. Y. *et al.* Epidemiology, management, and risk-adjusted mortality of ICU-acquired enterococcal bacteremia. *Clin. Infect. Dis.* **61**, 1413–1420 (2015).
41. Ong, D. S. Y. *et al.* Cytomegalovirus reactivation and mortality in patients with acute respiratory distress syndrome. *Intensive Care Med.* **42**, 333–341 (2016).
42. Padmanabhan, R., Meskin, N. & Haddad, W. M. Closed-loop control of anesthesia and mean arterial pressure using reinforcement learning. *Biomed. Signal Process. Control* **22**, 54–64 (2015).
43. Padmanabhan, R., Meskin, N. & Haddad, W. M. Reinforcement learning-based control for combined infusion of sedatives and analgesics. in *2017 4th International Conference on Control, Decision and Information Technologies (CoDIT)* 505–509 (IEEE, 2017).
44. Peine, A. *et al.* Development and validation of a reinforcement learning algorithm to dynamically optimize mechanical ventilation in critical care. *npj Digit Med* **4**, (2021).
45. Peng, C. *et al.* Impact of Early Tracheostomy on Clinical Outcomes in Trauma Patients Admitted the to Intensive Care Unit: A Retrospective Causal Analysis. *J. Cardiothorac. Vasc. Anesth.* (2022) doi:10.1053/j.jvca.2022.12.022.
46. Peng, X. *et al.* Improving Sepsis Treatment Strategies by Combining Deep and Kernel-Based Reinforcement Learning. *AMIA ... Annu. Symp. proceedings. AMIA Symp.* **2018**, 887–896 (2018).
47. Petersen, B. K. *et al.* Deep Reinforcement Learning and Simulation as a Path Toward Precision Medicine. *J. Comput. Biol. a J. Comput. Mol. cell Biol.* **26**, 597–604 (2019).
48. Pisani, M. A., Araujo, K. L. B. & Murphy, T. E. Association of cumulative dose of haloperidol with next-day delirium in older medical ICU patients. *Crit. Care Med.* **43**, 996–1002 (2015).
49. Pouwels, K. B. *et al.* Does appropriate empiric antibiotic therapy modify intensive care unit-acquired Enterobacteriaceae bacteraemia mortality and discharge? *J. Hosp. Infect.* **96**, 23–28 (2017).
50. Pouwels, K. B. *et al.* Intensive care unit (ICU)-acquired bacteraemia and ICU mortality and discharge: addressing time-varying confounding using appropriate methodology. *J. Hosp. Infect.* **99**, 42–47 (2018).

51. Pouwels, K. B. *et al.* Estimating the Effect of Healthcare-Associated Infections on Excess Length of Hospital Stay Using Inverse Probability-Weighted Survival Curves. *Clin. Infect. Dis.* **71**, E415–E420 (2020).
52. Prasad, N., Cheng, L.-F., Chivers, C., Draugelis, M. & Engelhardt, B. E. *A reinforcement learning approach to weaning of mechanical ventilation in intensive care units*. *Preprint at* https://arxiv.org/abs/1704.06300 (2017).
53. Prasad, N. *et al.* Guiding Efficient, Effective, and Patient-Oriented Electrolyte Replacement in Critical Care: An Artificial Intelligence Reinforcement Learning Approach. *J. Pers. Med.* **12**, (2022).
54. Raghu, A., Komorowski, M. & Celi, L. A. Continuous state-space models for optimal sepsis treatment: a deep reinforcement learning approach. in *Machine Learning for Healthcare Conference* (2017).
55. Raghu, A., Komorowski, M., Ahmed, I. & Celi, L. *Deep reinforcement learning for sepsis treatment*. *Preprint at* https://arxiv.org/abs/1711.09602 (2017).
56. Ribba, B., Bräm, D. S., Baverel, P. G. & Peck, R. W. Model enhanced reinforcement learning to enable precision dosing: A theoretical case study with dosing of propofol. *CPT Pharmacometrics Syst. Pharmacol.* **11**, 1497–1510 (2022).
57. Roggeveen, L. *et al.* Transatlantic transferability of a new reinforcement learning model for optimizing haemodynamic treatment for critically ill patients with sepsis. *Artif. Intell. Med.* **112**, (2021).
58. Shahn, Z., Shapiro, N. I., Tyler, P. D., Talmor, D. & Lehman, L.-W. H. Fluid-limiting treatment strategies among sepsis patients in the ICU: A retrospective causal analysis. *Crit. Care* **24**, (2020).
59. Shahn, Z., Lehman, L.-W. H., Mark, R. G., Talmor, D. & Bose, S. Delaying initiation of diuretics in critically ill patients with recent vasopressor use and high positive fluid balance. *Br. J. Anaesth.* **127**, 569–576 (2021).
60. Shahn, Z. *et al.* Effects of aggressive and conservative strategies for mechanical ventilation liberation. *J. Crit. Care* **76**, 154275 (2023).
61. Sinzinger, E. D. & Moore, B. Sedation of simulated ICU patients using reinforcement learning based control. *Int. J. Artif. Intell. Tools* **14**, 137–156 (2005).
62. Steen, J. *et al.* Attributable mortality of ventilator-associated pneumonia replicating findings, revisiting methods. *Ann. Am. Thorac. Soc.* **18**, 830–837 (2021).
63. Su, L. *et al.* Establishment and Implementation of Potential Fluid Therapy Balance Strategies for ICU Sepsis Patients Based on Reinforcement Learning. *Front. Med.* **9**, 1–14 (2022).
64. Tacquard, C. *et al.* Impact of High-Dose Prophylactic Anticoagulation in Critically Ill Patients With COVID-19 Pneumonia. *Chest* **159**, 2417–2427 (2021).
65. Torres, L. K. *et al.* Attributable mortality of acute respiratory distress syndrome: A systematic review, meta-analysis and survival analysis using targeted minimum loss-based estimation. *Thorax* (2021) doi:10.1136/thoraxjnl-2020-215950.
66. Truche, A.-S. *et al.* Continuous renal replacement therapy versus intermittent hemodialysis in intensive care patients: impact on mortality and renal recovery. *Intensive Care Med.* **42**, 1408–1417 (2016).
67. Urner, M. *et al.* Venovenous extracorporeal membrane oxygenation in patients with acute covid-19 associated respiratory failure: comparative effectiveness study. *BMJ* **377**, 1–9 (2022).

68. Wang, W. *et al.* Estimating the Causal Effect of Low Tidal Volume Ventilation on Survival in Patients with Acute Lung Injury. *J R Stat Soc Ser C Appl Stat* **60**, 475–496 (2011).
69. Wang, Y., Zhao, Y. & Petzold, L. Predicting the need for blood transfusion in intensive care units with reinforcement learning. in *Proceedings of the 13th ACM International Conference on Bioinformatics, Computational Biology and Health Informatics* 1–10 (2022).
70. Wang, Z., Zhao, H., Ren, P., Zhou, Y. & Sheng, M. Learning Optimal Treatment Strategies for Sepsis Using Offline Reinforcement Learning in Continuous Space. in *International Conference on Health Information Science* 113–124 (Springer, 2022).
71. Weng, W.-H., Gao, M., He, Z., Yan, S. & Szolovits, P. *Representation and reinforcement learning for personalized glycemic control in septic patients*. *Preprint at* https://arxiv.org/abs/1712.00654 (2017).
72. Yang, Z. *et al.* Optimization of Dry Weight Assessment in Hemodialysis Patients via Reinforcement Learning. *IEEE J. Biomed. Heal. Informatics* **26**, 4880–4891 (2022).
73. Yarnell, C. J. *et al.* Oxygenation thresholds for invasive ventilation in hypoxemic respiratory failure: a target trial emulation in two cohorts. *Crit. Care* **27**, 67 (2023).
74. Zhang, L. *et al.* The Role of Glucocorticoids in the Treatment of ARDS: A Multicenter Retrospective Study Based on the eICU Collaborative Research Database. *Front. Med.* **8**, (2021).
75. Zhang, Q., Wang, J., Liu, G. & Zhang, W. Artificial intelligence can use physiological parameters to optimize treatment strategies and predict clinical deterioration of sepsis in ICU. *Physiol. Meas.* **44**, (2023).
76. Zhang, R. *et al.* The Effect of Loop Diuretics on 28-Day Mortality in Patients With Acute Respiratory Distress Syndrome. *Front. Med.* **8**, (2021).
77. Zhang, Z., Zhu, C., Mo, L. & Hong, Y. Effectiveness of sodium bicarbonate infusion on mortality in septic patients with metabolic acidosis. *Intensive Care Med.* **44**, 1888–1895 (2018).
78. Zhang, Z., Mo, L., Ho, K. M. & Hong, Y. Association between the use of sodium bicarbonate and mortality in acute kidney injury using marginal structural COx model. *Crit. Care Med.* **47**, 1402–1408 (2019).
79. Zheng, H., Zhu, J., Xie, W. & Zhong, J. Reinforcement learning assisted oxygen therapy for COVID-19 patients under intensive care. *BMC Med Inf Decis Mak* **21**, 350 (2021).
